# Farnesoid X receptor: From Structure to Function and Its Pharmacology in Liver Fibrosis

**DOI:** 10.14336/AD.2023.0830

**Published:** 2024-08-01

**Authors:** Chuan Ding, Zeping Wang, Xinyue Dou, Qiao Yang, Yan Ning, Shi Kao, Xianan Sang, Min Hao, Kuilong Wang, Mengyun Peng, Shuosheng Zhang, Xin Han, Gang Cao

**Affiliations:** ^1^School of Pharmacy, Zhejiang Chinese Medical University, Hangzhou, China.; ^2^College of Chinese Materia Medica and Food Engineering, Shanxi University of Chinese Medicine, Jinzhong, China.; ^3^Jinhua Institute, Zhejiang Chinese Medical University, Jinhua, China.

**Keywords:** FXR, liver fibrosis, structure, pharmacology, clinical investigations

## Abstract

The farnesoid X receptor (FXR), a ligand-activated transcription factor, plays a crucial role in regulating bile acid metabolism within the enterohepatic circulation. Beyond its involvement in metabolic disorders and immune imbalances affecting various tissues, FXR is implicated in microbiota modulation, gut-to-brain communication, and liver disease. The liver, as a pivotal metabolic and detoxification organ, is susceptible to damage from factors such as alcohol, viruses, drugs, and high-fat diets. Chronic or recurrent liver injury can culminate in liver fibrosis, which, if left untreated, may progress to cirrhosis and even liver cancer, posing significant health risks. However, therapeutic options for liver fibrosis remain limited in terms of FDA-approved drugs. Recent insights into the structure of FXR, coupled with animal and clinical investigations, have shed light on its potential pharmacological role in hepatic fibrosis. Progress has been achieved in both fundamental research and clinical applications. This review critically examines recent advancements in FXR research, highlighting challenges and potential mechanisms underlying its role in liver fibrosis treatment.

## 1. Introduction

Farnesoid X receptors (FXRs) belong to the nuclear receptor superfamily and are primarily expressed in the liver, small intestine, and kidneys [1]. Due to their roles in regulating the homeostasis of bile acids (BAs) and glucose lipids, FXRs are progressively becoming attractive targets for research and drug development [2]. Studies have demonstrated that FXRs consist of two members in mammals: FXRα (NR1H4) and FXRβ (NR1H5) [3]. Among these, FXRβ can perform receptor functions in certain species, such as rabbits, rats, dogs, and mice, while it encodes a pseudogene in primates and humans [4]. In contrast to FXRβ, the FXRα gene encodes four biologically active subtypes (α1, α2, α3, and α4), with their expression exhibiting tissue-dependent patterns [5]. FXR regulates the transcription process of target genes by binding to the FXR response element, either through the formation of FXR-RXR heterodimers in most cases or FXR monomers in rare cases [6, 7]. Microbiota, enterohepatic circulation, and liver illnesses represent only a few examples of metabolic and immunological diseases in which FXR, a transcription factor, can be activated by ligand binding [8, 9]. Notably, FXR also serves as a multifunctional cytoprotective agent and tumor inhibitor in the liver, holding significant importance for the amelioration of liver injuries [10-12]. Furthermore, liver fibrosis resulting from chronic liver injury poses a risk factor for cirrhosis or liver cancer, significantly endangering human health. However, the treatment of liver fibrosis encounters formidable challenges due to limitations in available therapeutic drugs. Characterized by the excessive deposition of extracellular matrix (ECM) and excessive proliferation, liver fibrosis refers to a group of clinical and pathological syndromes accompanied by abnormal liver structure and function [13]. Its underlying nature is a tissue compensatory response secondary to liver inflammation or injury [14]. During the course of chronic liver disease, hepatocytes undergo repetitive destruction and regeneration, leading to the manifestation of extracellular matrices marked by elevated expression of collagen, glycoproteins, and proteoglycans. These components become abnormally distributed within the liver, culminating in the formation of scars [15]. Numerous factors associated with prolonged chronic stimulation can trigger inflammation and necrosis of hepatocytes, thereby promoting the activation of hepatic stellate cells (HSCs) and resulting in hepatic fibrosis [16, 17]. For instance, liver fibrosis arises in the context of various conditions, including viral hepatitis, schistosomiasis infections, alcoholic or nonalcoholic fatty liver disease (NAFLD), Wilson's disease, drug-induced or toxic liver diseases, cholestasis, and autoimmune liver diseases [18, 19]. Common clinical manifestations observed in patients with hepatic fibrosis include fatigue, loss of appetite, abnormal stool, discomfort, swelling, pain in the liver area, and a dark complexion [15]. Some patients may also exhibit additional clinical manifestations that coincide with the underlying disease [20]. Left untreated, hepatic fibrosis can progress to cirrhosis or even hepatocellular carcinoma (HCC). According to the 2020 Global Cancer Survey data, primary liver cancer-induced HCC accounts for up to 830,000 deaths, making it the third leading cause of cancer-related mortality. This reality has become a significant impediment to the well-being of individuals in numerous countries [21]. Research has demonstrated that the growth and progression of liver cancer are intimately linked with the microenvironment influenced by interstitial cells involved in liver fibrosis [22]. Consequently, there is an urgent need to explore therapeutic agents for liver fibrosis. Currently, FXR activation is implicated in various cellular molecular pathways, including hepatocyte protection and the inhibition of HSC activation, displaying a promising antifibrotic effect [23]. Importantly, obeticholic acid, an FXR agonist, has entered clinical trials as a potential antifibrosis drug [24, 25].

Building upon these findings, this article presents a comprehensive review of the structure, function, and pharmacology of FXR, with a particular focus on its pharmacological effects in liver fibrosis. Furthermore, the article offers strategies to enhance the prevention and treatment of liver fibrosis.

**Figure 1.**
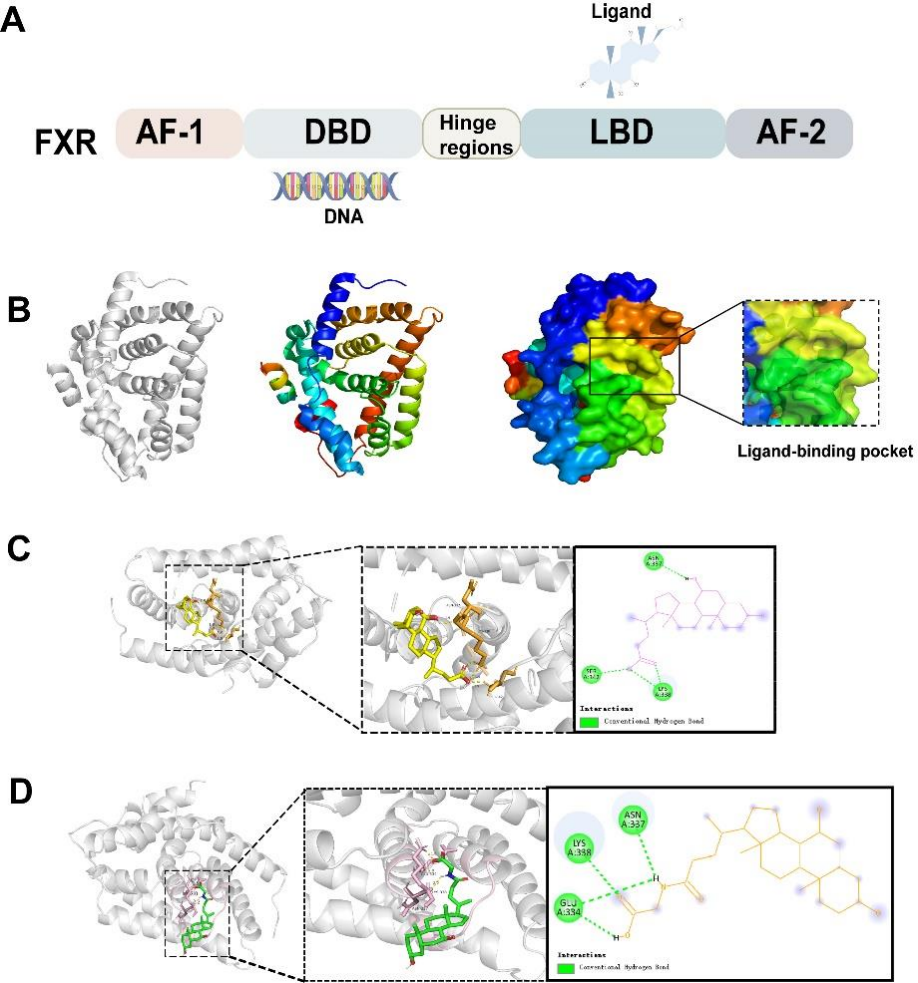
**Three different crystal form structures known to FXR (PDB ID is 4QE6)**. (**A**) Common architecture of FXR. (**B**) The approximate location of the apo-FXR and its bonded pocket that employs different conformation according to the species of ligands. (**C**) Activated conformation of FXR combined with CDCA (agonist-FXR). (**D**) Antagonistic conformation of FXR binding to GUDCA (antagonist-FXR).

## 2. FXR structure and function

FXR, with a typical nuclear receptor structure, could regulate key metabolic and immune-related processes, such as BA homoeostasis, glucose lipid metabolism and energy consumption, as well as the inflammatory response, cellular proliferation and fibrosis [26], which are explained in detail as follows.

### 2.1. Structure of FXR and its crystalline structure with ligand complexes

FXR was initially identified and named in 1995 after the discovery of its activation by farnesol and its metabolites [27, 28]. Devarakonda and colleagues indicated that FXR comprises nonligand-dependent transcriptional activation domains (AF-1), DNA binding domains, hinge regions, ligand binding domains (LBD), and carboxyl terminals containing ligand-dependent activation domains (AF-2) [3] (Fig 1A). In fact, the structure of FXR's LBD closely resembles that of other members within the nuclear receptor family. As early as 2002, the X-ray crystal structure of HNF4α's LBD at high resolution unveiled the presence of fatty acids within the binding pocket, along with the AF2 helix adopting a conformation characteristic of a transcriptionally active nuclear receptor [29]. Subsequently, Sladek highlighted that the true ligand binds within a hydrophobic pocket of the LBD, prompting the repositioning of AF-2. This in turn induces conformational changes that activate co-suppressors and introduce coactivators [30]. Regarding FXR activation, ligands bind to the LBD region of FXR, inducing diverse dynamic conformational changes and orientational shifts of AF-2. These alterations collectively regulate downstream target gene transcription through FXR-RXR heterodimers or FXR monomers, thereby participating in metabolic homeostasis [31, 32]. Moreover, FXR features a flexible ligand-binding pocket within its LBD that adopts distinct conformations based on the ligand species [33]. Jiang and collaborators indicated that structures of FXR-ligand complexes in the PDB database can be broadly categorized into three types: FXR without ligand binding (apo-FXR) (Fig 1B), FXR bound to agonists (agonist-FXR) (Fig 1C), and FXR bound to antagonists (antagonist-FXR) (Fig 1D) [34]. Drawing upon the functional and structural aspects of FXR, numerous research laboratories worldwide, particularly within the pharmaceutical industry, have been dedicated to developing ligands targeting FXR. Recent studies have achieved some headway in seeking drugs that target FXR for the treatment of metabolic diseases. Notably, advancements have been made in both basic and clinical realms. However, given that the liver serves as the hub of human metabolism and that there are limited FDA-approved drugs for liver fibrosis therapy, we present an overview of recent advancements in the development of FXR regulators. This includes exploration of endogenous, exogenous, and naturally allosteric compounds as potential treatments for liver fibrosis. Our approach considers the structural and functional attributes of FXR.

#### 2.1.1. Endogenous regulators

As we mentioned earlier, primary BAs are synthesized directly by hepatocytes and mainly include CA, CDCA, and taurocholate acid. Secondary BAs, on the other hand, are generated through the breakdown of intestinal bacteria and the intestine-liver circulation, predominantly including DCA, lithocholic acid (LCA), and GUDCA [35, 36]. CDCA stands out as the most potent ligand for FXR, succeeded by CA, DCA, and LCA, with their efficacy being closely tied to their structural features [37]. In the context of metabolic diseases, there is an upregulation of FXR antagonistic DCA in serum, coupled with a downregulation of CDCA (an FXR agonist) [38]. Specifically, DCA has the capacity to incite intestinal inflammation [39], while CDCA activates intestinal FXRs and ameliorates glucose metabolism associated with polycystic ovary syndrome [40]. However, the oral administration of CDCA does not restore plasma bile acid levels to normal levels; instead, it leads to elevated supraphysiological concentrations of endogenous CDCA and its derivatives [41]. Furthermore, FXR antagonists such as GUDCA and TUDCA exhibit the ability to mitigate metabolic disorders in obese mice [42, 43]. Intriguingly, LCA, an FXR antagonist possessing partial agonist activity, can induce cholestatic pruritus or reduce the expression of bile salt export pump (BSEP) under varying physiological conditions [44, 45]. Endogenous BAs display limited selectivity for FXR, which is a key reason for their limited usage in the treatment of liver diseases. Nonetheless, they offer a valuable framework for the synthesis of novel and selective FXR ligands, thereby serving as a promising scaffold for further research and development.

#### 2.1.2. Synthetic regulators

Given FXR's pivotal role in metabolic diseases, high-throughput strategies and structure-oriented drug development have been employed to screen synthetic ligands. Clinical trials have demonstrated that compared to a placebo, obeticholic acid (OCA), a semisynthetic derivative of CDCA, effectively ameliorates the histological and biochemical characteristics of nonalcoholic steatohepatitis (NASH) without cirrhosis [46]. Additionally, OCA leads to a reduction in sodium+/taurocholate cotransporting polypeptide, influencing BA circulation and subsequently lowering the expression of α-SMA in human HSCs, thereby attenuating liver fibrosis [47]. Among 141 patients treated with OCA, approximately 33 (23%) experienced pruritus (p < 0.0001). Further exploration is warranted to understand this phenomenon. As a full non-BA agonist of FXR, GW4064, notable for its high potency and selectivity, significantly decreases serum cholesterol and triglyceride levels in individuals with NAFLD. Unfortunately, it failed to progress beyond phase I clinical trials due to issues of instability [48]. Through structural modifications to GW4064, stable and pharmacologically viable FXR agonists have been developed, such as cilofexor and tropifexor. These compounds exhibit therapeutic effects on liver fibrosis in NASH patients [49, 50]. Furthermore, research on partial agonists of FXR is in active progress. For instance, fexaramine, an intestinal FXR agonist, has shown promise in reducing systemic inflammation and improving metabolism in obese mice [51]. Nidufexor, a partial FXR agonist, has demonstrated marked effects in patients with NASH [52]. We have summarized partially synthesized FXR ligands to establish a foundation for subsequent investigations. The regulatory factors and related pharmacological activities of these partially synthesized FXR ligands have been consolidated in Table 1, contributing to the establishment of a framework for future studies.

**Table 1:** Partial FXR ligands.

	Types	Name	Outcomes	Refs.
**Endogenous**	Agonist	Androsterone	directly bind to purified hFXR ligand-binding domain (LBD) protein, recruit steroid receptor coactivator protein-1 (SRC-1) coactivator peptide	[235]
		CDCA	improve glucose metabolism and increase the mRNA expression of BSEP	[40, 44]
	Antagonist	TUDCA	improve glucose metabolism	[43]
		5Alpha-bile alcohols	modulate intestinal lipid absorption and expression of genes involved in the biosynthesis/catabolism of BAs	[236]
		T-βMCA	reduce the TCA-induced expression of *FGF 15 i*n the ileum	[237]
		GUDCA	improve various metabolic endpoints in mice with obesity	[42]
		DCA	promote intestinal inflammation	[238]
	Selective modulators	progesterone metabolite, epiallopregnanolone sulfate	reduce FXR-mediated discharge of BAs and secretion of FGF19.	[239, 240]
		lithocholic acid (LCA)	a hydrophobic bile acid and strongly decrease BSEP	[44]
**Synthetic**	Agonist Agonist	GW4064	completely restores impaired BA way and metabolic syndrome in iron-fed mice.	[241]
		INT-767	relieves podocyte injury, mesangial expansion, collagen deposition and tubulointerstitial fibrosis	[214, 242]
		SU5	regulate lipid metabolism and triglyceride metabolism	[243]
		OCA	alleviated the histological and biochemical features of NASH without cirrhosis	[46]
		6α-ethyl-24-norcholanyl-23-amine	behave as full FXR agonist endowed with high binding affinity and efficacy	[43]
		fexaramine	reduces systemic inflammation and metabolic improvement in obese mice	[51]
		HEC96719	show higher FXR selectivity and more favorable tissue distribution dominantly in liver and intestine	[244]
		Nidufexor (LMB763)	regulates FXR-dependent gene in vitro and in vivo	[52]
		BAR502	reverses steatohepatitis and fibrosis caused by chronic exposure of mice to a high caloric diet	[245]
		tropifexor (LJN452)	reverses developed fibrosis, reduces non-alcoholic fatty liver disease activity scores and liver triglycerides	[246]
	Antagonist	3-(tert-Butyl)-4-hydroxyphenyl	obtain antagonistic activity of FXR	[247]
		4-({1-[5-({[1-tert-butyl-5-(4-fluorophenyl)-1H-pyrazol-4-yl]carbonyl}amino)-2-chlorobenzyl]piperidin-4-yl}oxy)benzoic acid	induce remarkable beneficial changes in both plasma non-HDL-cholesterol and HDL-cholesterol levels	[248]
		9,11-seco-cholesterol derivatives	display the best FXR antagonistic activity at the cellular level and decrease the target genes of FXR	[249]
		FLG249	controls the level of FXR target gene in mouse ileum	[250]
**Natural**	Agonist	Farnesol	stimulate growth of MCF-7 breast cancer cell	[251]
		20*S*-protopanaxatriol	ameliorated hepatic inflammation and fibrosis induced by TAA	[252]
		alisol A 23-acetate	antihyperglycemic	[253]
		alisol B 23-acetate	protects against ischemic acute kidney injury (AKI)	[254]
		altenusin	Attenuates NAFLD by reducing the body weight and fat mass	[255]
		Auraptene	Liver protection, anti-inflammatory and antioxidant activities	[243]
		berberine	reducing hepatic gluconeogenesis and lowering blood glucose	[256]
		Calycosin	improve liver steatosis and reduce liver fibrosis	[257]
		Hedragonic acid	protect mice from liver injury induced by acetaminophen overdose and decrease hepatic inflammatory responses	[258]
		dihydroartemisinin	attenuate portal hypertension by targeting HSC contraction	[259]
		hesperidin	Prevent cholestatic liver injury	[260]
		isotschimgine	alleviates nonalcoholic steatohepatitis and fibrosis	[261]
		schaftoside	could attenuate APAP-induced hepatotoxicity *by* regulating oxidative stress and inflammation	[262]
		Gypenosides	ameliorated NASH	[263]
		Swertiamarin	alleviate cholestasis	[264]
		conicasterol E	triggers BA detoxification	[265]
		cycloastragenol	Reduce fatty diet-induced liver lipid accumulation	[266]
		Ginsenoside Rc	Relieve inflammation and oxidative stress	[267]
		Cafestol	increased fat oxidation and energy expenditure	[268]
		coumestrol	exert beneficial effects on lipid and glucose metabolism	[269]
	Antagonist	Naringin	promoting BA synthesis from cholesterol by upregulating CYP7A1	[270]
		scalarane sesterterpenes	Inhibition of the interaction between FXR and SRC-1	[271]
		SIPI-7623	decreased the level of cholesterol and triglyceride	[272]
		Stigmasterol	contribute to BA-induced hepatocyte damage	[273]
		sulfated sterol	Inhibit a subset of FXR regulatory genes in hepatocytes	[274]
		guggulsterone	Reduced liver cholesterol in wild-type mice fed a high cholesterol diet, but not in FXR-deficient mice	[275]
	selective modulators	Oleanolic acid	reduce the biosynthesis of BAs and the cytotoxicity caused by the increase of BAs	[276]

#### 2.1.3. Natural regulators

As the structural investigation of FXR agonists progresses, the presence of FXR regulators in natural products is gradually coming to light. These regulators encompass glycosides, flavonoids, and polyphenols. Salidroside, the primary active ingredient found in the plant Rhodiola rosea, exhibits the capacity to enhance NAFLD through the microbiota-BA-FXR axis and the AMPK-dependent TXNIP/NLRP3 pathway [53, 54]. Another natural isoflavone compound, formononetin, enhances liver/systemic BA metabolism and mitigates liver injury by modulating the SIRT1-FXR pathway [55]. Similarly, curcumin, a polyphenol, mitigates cholestasis by affecting BA and inflammation, both of which are regulated by FXR [56]. Furthermore, epigallocatechin gallate, a polyphenol found in green tea, has the capability to activate FXR, with its effectiveness being concentration-dependent [57]. Numerous other compounds present in natural products also possess the ability to regulate FXR. Table 1 compiles a summary of these regulators and their associated pharmacological effects in certain natural products. The aim is to contribute to the clinical advancement of natural products with potential FXR-modulating properties.

### 2.2. Functions of FXR

FXR, functioning as a BA receptor, becomes activated by specific BA metabolites and operates as a transcription factor [2]. Its role is intricately linked to the regulation of diverse physiological processes, encompassing BA synthesis, enterohepatic circulation, glucose, and lipid metabolism [58]. In a study by Sun et al., the potential impact of FXR on rectal cancer, liver cancer, and other metabolic diseases were comprehensively summarized [58]. In this update, we will cover the latest advancements concerning FXR in the realms of BA and the intestine. Additionally, we will delve into its involvement in enterohepatic circulation and gut-to-brain communication, with a specific focus on its relevance in liver fibrosis and the potential for targeted drug development.

**Figure 2.**
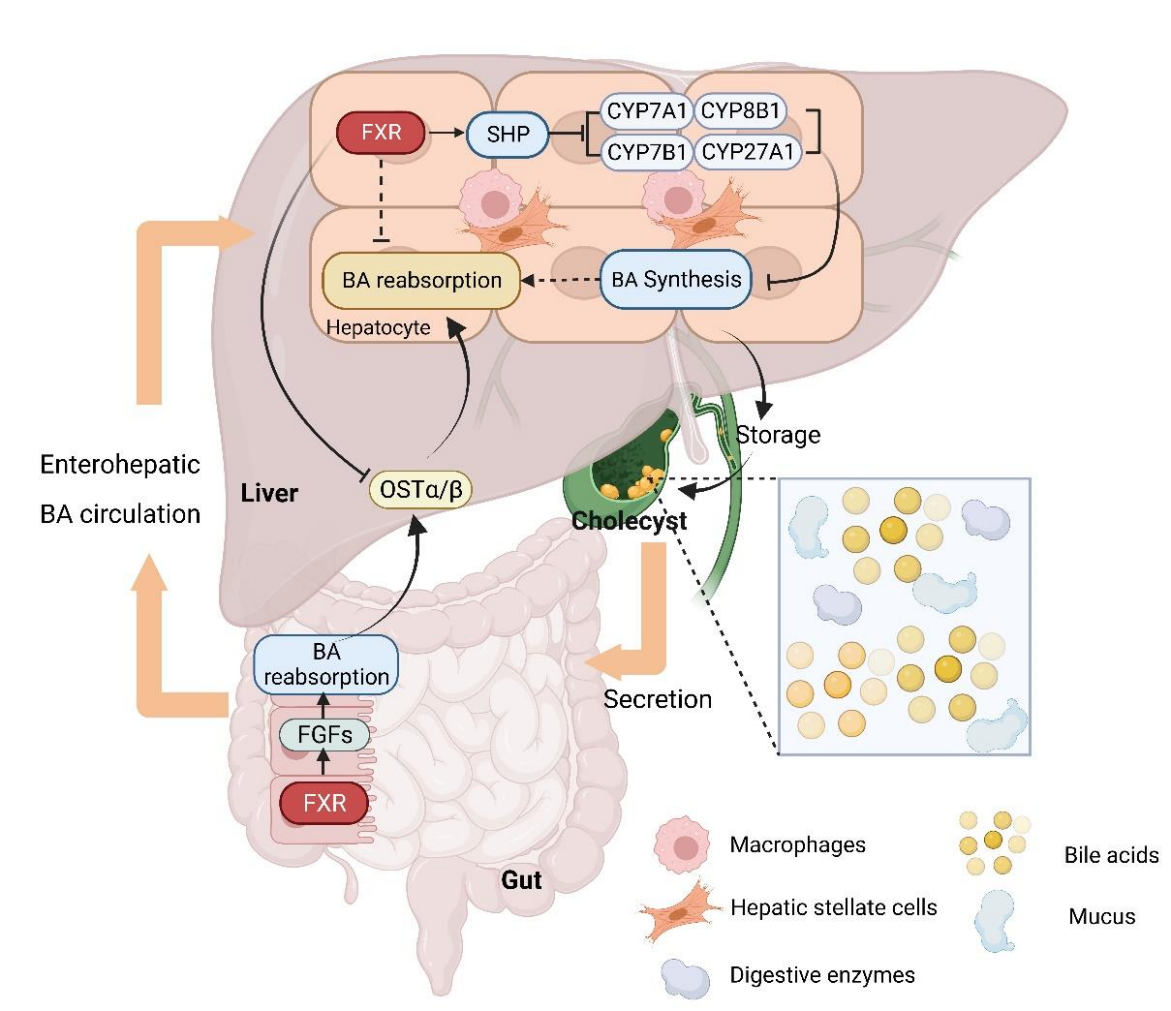
**Relationship between FXR and BAs**. BAs are mainly synthesized by hepatocytes via the classical pathway or alternative pathway, all of which are regulated by FXRs expression. Activated FXRs can reduce the expression of BA synthases through SHP or FGFs. The relationship between FXR and BAs is mainly established through hepatoenteric circulation.

#### 2.2.1. The relationship between FXR and BAs

BAs are steroid compounds synthesized by hepatocytes from cholesterol and play a role in the digestion and absorption of lipids. Jia and colleagues have elucidated that BA synthesis in the liver follows two distinct pathways: the classical pathway and the alternative pathway [59]. The classical pathway, which accounts for 75% of BA synthesis, is initiated by cholesterol 7α-hydroxylation through the catalysis of cholesterol 7-alpha hydroxylase (CYP7A1). Subsequently, the steroid nucleus undergoes transformation, and its side chain undergoes oxidative cleavage, which is regulated by CYP8B1 [59]. On the other hand, the alternative pathway, known as the acidic pathway, is instigated by cholesterol 27-hydroxylation via CYP27A1. The resulting oxysterol products are then subjected to further hydroxylation via catalysis of oxysterol 7α-hydroxylase (CYP7B1) [59]. Research has indicated that non-20α-hydroxylated (chenodeoxycholic acid) synthesized via the alternative pathway exerts positive effects on lipid, cholesterol, and glucose metabolism. In cases where these processes become aberrant, the proportion of 12-hydroxy bile acid content synthesized through the classical pathway tends to increase, leading to a reduction in the control capacity over lipid homeostasis. This, in turn, can escalate inflammation and fibrosis within the liver [60, 61]. Clinical studies have demonstrated significantly elevated levels of serum and liver BAs in patients with liver fibrosis. Delving into BA-related signaling pathways might hold promise as a prospective avenue for liver disease treatment [62]. Recent findings have unveiled the involvement of several nuclear receptors in the metabolic homeostasis of BAs, with FXR standing out as a crucial member [63-65], often referred to as a BA receptor [66]. FXR primarily regulates BAs by modulating their reabsorption through various mechanisms. For instance, FXR can reduce the expression of BA synthases through the regulation of the short heterodimer partner or ﬁbroblast growth factor (FGF) pathways. Additionally, activated FXR can dampen the expression of BA uptake carriers, thereby inhibiting liver reabsorption. Furthermore, FXR can stimulate bile secretion and BA reabsorption by directly binding to the response element of BA secretion carriers or by regulating the expression of intestinal BA binding protein [67, 68]. Of notable importance is the fact that FXR is prominently distributed at sites where BAs exert their effects, with the highest concentration being in the liver, followed by the small intestine and kidneys [69]. As a result, the negative feedback regulatory influence of FXR on BAs predominantly operates through the intestinal and hepatic circulation (Fig. 2) [70].

**Figure 3.**
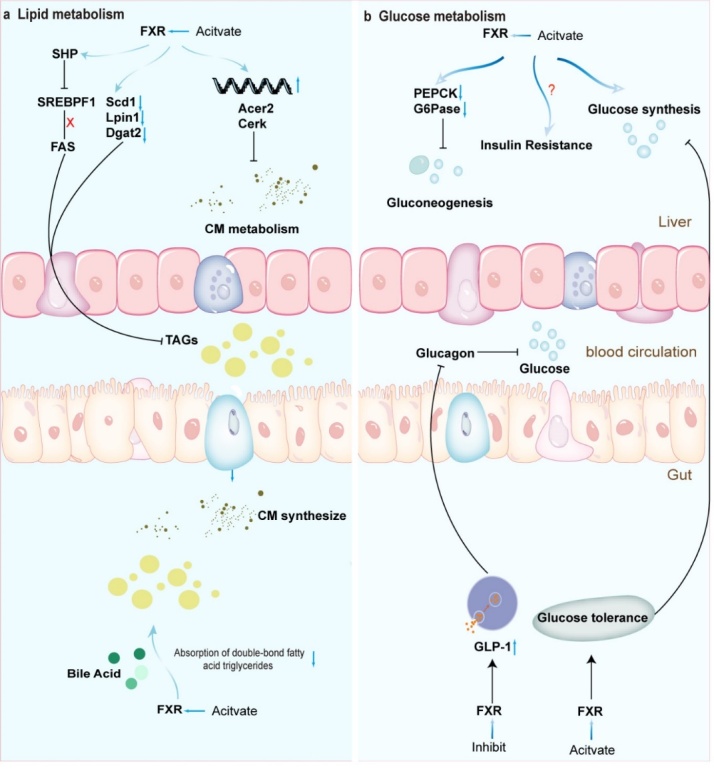
**FXR in glucose lipid metabolism**. In lipid metabolism, FXR reduces liver fat by inhibiting fat formation and promoting fatty acid oxidation; these functions are related to the expression of SREBP1 or PPARs, respectively; in glucose metabolism, FXR participates in glucose homeostasis through two pathways, namely, gluconeogenesis-related genes and glucagon-activated factors.

#### 2.2.2. FXR in glucose lipid metabolism

FXR's influence extends beyond the realm of BA metabolism, encompassing the maintenance of the body's glucose and lipid metabolism homeostasis (Fig. 3). Watanabe and colleagues proposed that FXR diminishes de novo liver fat accumulation through the FXR-SHP-SREBP1c pathway, thus leading to a reduction in liver triglycerides [71]. A recent comprehensive lipid omics analysis revealed that FXR activation lowers hepatic levels of polyunsaturated and monounsaturated fatty acids (PUFAs and MUFAs) through two distinct pathways. These pathways involve the inhibition of lipogenesis gene expression and lipid absorption [72]. Specifically, FXR curtails MUFA levels by inhibiting Dgat2, Lpin1, and Scd1 expression. Notably, this effect operates independently of SHP and SREBP1. In contrast, FXR's effect on PUFA reduction is primarily mediated through the suppression of lipid absorption [72]. Further investigations have demonstrated that FXR is intricately involved in the transcriptional-level expression of miR-552-3p, which governs metabolic genes in glycolipid metabolic disorders through cis-binding [73]. In the context of glucose metabolism regulation, hepatic FXR positively modulates glucose production through two mechanisms. One mechanism entails protein kinase A-mediated FXR phosphorylation, contributing to the activation of gluconeogenic genes by activated FXR and cAMP-response element-binding protein. The second mechanism involves the suppression of FXR's anti-gluconeogenic function through FOXA2, which can be activated by glucagon. Particularly noteworthy is the physical interaction between FOXA2 and FXR. It is worth mentioning that low expression of Foxa2 does not affect the expression of gluconeogenic genes induced by glucagon. However, FXR agonists enhance the expression of gluconeogenic genes, indicating that the protein kinase A and FOXA2 pathways are involved in distinct FXR-mediated glucose metabolism pathways [74]. Moreover, bile transfer to the ileum enhances glucose homeostasis via the intestinal FXR-GLP-1 pathway. In particular, during fasting, BA signaling through intestinal FXR enhances intestinal GLP-1 production, which promotes intestinal glucose tolerance by augmenting insulin secretion [75]. In light of this, Zhang and colleagues injected taurocholate into the midjejunum of healthy men, resulting in increased expression of GLP-1 and insulin secretion, ultimately upregulating energy uptake by the large intestine [76]. Additionally, intestinal FXR exerts control over hepatic gluconeogenesis by influencing pyruvate carboxylase and mitochondrial acetyl-CoA levels [77]. FXR's influence on glycolipid metabolism does not solely rely on its pharmacological effects but is also intricately linked to the intestinal microbiota. FXR is involved in the metformin-regulated gut microbiome in the context of metabolic dysfunction, operating via the *B. fragilis*-glycoursodeoxycholic acid-intestinal FXR axis [42]. Given its multifaceted functions, current studies highlight the potential of targeted FXR activation as a more reliable approach compared to systemic therapies.

#### 2.2.3. FXR with microbiota

**Figure 4.**
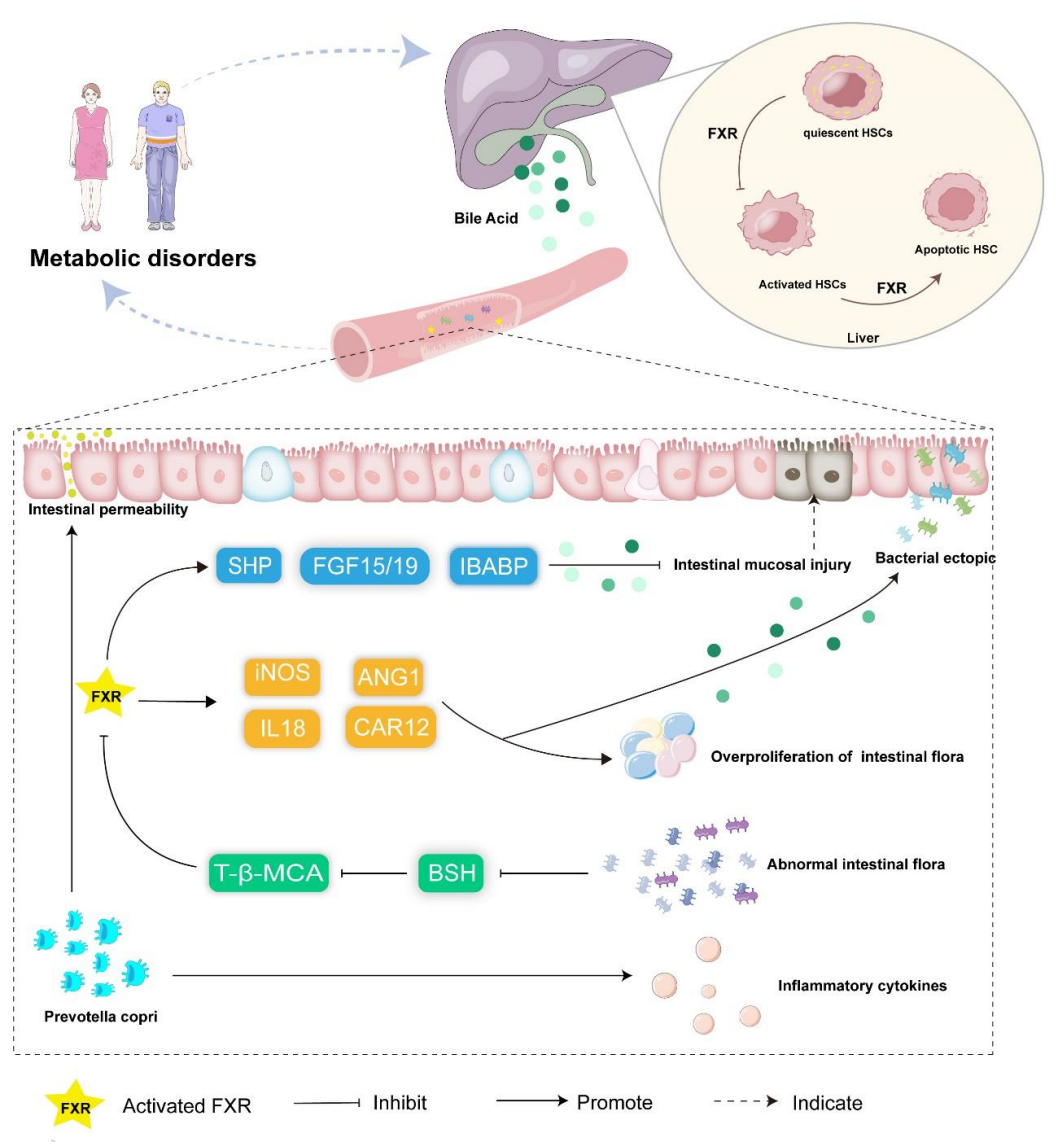
**Overview of FXRs’ regulation of intestinal flora homeostasis and metabolic disorders**. The imbalance between the body and the intestinal flora leads to the disturbance of normal physiological functions. The intestinal flora promotes fatty inflammation, steatosis, and obesity in an FXR-dependent manner. The activation of intestinal FXR can inhibit the abnormal proliferation of bacteria and reduce intestinal mucosal damage.

Based on sequence analysis results, the human intestine is host to a range of 1000 to 1150 bacterial species, categorized into 7 bacterial phyla: Pachylobacteria, Bacteroides, Actinomycetes, Proteus, Clostridium, Wartymic microbacteria, and Cyanobacteria. Notably, Pachylobacteria and Bacteroides together constitute over 95% of this microbial community [78-80]. The equilibrium between the human body and the intestinal microbiota, which is essential for normal physiological functions such as immunity, metabolism, and inflammation, can be disrupted by various factors, including chronic diseases and medications [81-83]. A study by Jian et al. indicated that abdominal irradiation prompts alterations in gut microbiota composition, leading to decreased abundance and diversity [84]. They further elucidated the role of L. plantarum in activating the FXR-fibroblast growth factor 15 (FGF15) signaling pathway, which promotes DNA damage repair in intestinal epithelial cells [84]. It has been observed that intestinal FXR activation can curb aberrant bacterial proliferation and mitigate intestinal mucosal damage [85]. Building upon this, FXR activators have been employed to attenuate pathological bacterial translocation via the portal-venous route [86, 87]. Ava Parséus and colleagues challenged the conventional genetic notion by discovering that the microbiota influences adipose inflammation, steatosis, and obesity in an FXR-dependent manner. To substantiate this, they transplanted microbiota from FXR-deficient mice and FXR-competent mice into germ-free mice, demonstrating that the altered microbiome partially ameliorated the metabolic status of FXR-deficient mice [88]. Considering the intricate interplay between FXR and the microbiota, Shu and team found that berberine alleviates NASH by regulating the interplay between the gut microbiome-manifested through increased relative abundance of Clostridium, Lactobacillus, and Phytodermidae- and BA metabolism, along with activating gut FXR [89]. Additionally, the microbiota can influence the activation of FXR by binding amino acids to bile acids [90] (Fig 4). Investigations have demonstrated that Pu-erh tea confers hypolipidemic effects by suppressing microorganisms linked to bile salt hydrolase activity, thereby diminishing FXR-FGF15 signaling [91]. While the correlation between the gut microbiota and FXR warrants further exploration, targeting the microbiome is emerging as a promising therapeutic avenue for preventing and treating gut-related disorders.

#### 2.2.4. FXR in enterohepatic circulation

The intestinal microbiota exerts an influence on inflammation and immunity, a relationship closely intertwined with hepato-intestinal circulation [92]. According to the theory of the entero-liver axis, the liver and intestine establish a symbiotic relationship marked by the liver's profound processing of microbiota metabolites and the regulation of microbiota structure through the secretion of metabolic substances, such as BAs [93]. As previously mentioned, BAs are synthesized in the liver and stored in the bile duct or gallbladder. Upon food intake, they are released into the small intestine, where they undergo further metabolism by gut bacteria. Subsequently, BAs are transported back into the enterohepatic circulation, with approximately 95% being reabsorbed in the ileum and entering the portal vein of the liver. They then traverse liver sinusoids and are conveyed to hepatic cells [94, 95]. Throughout these processes, liver FXR not only suppresses BA synthesis but also enhances the recirculation of BAs from the gut to the liver via the organic solute transporter alpha/beta (OSTα/β). Furthermore, it regulates bile acid intake through the portal vein circulation [96, 97]. Meanwhile, intestinal FXR activates downstream gene expression via the FGF 15/19 pathway, thereby curbing abnormal total BA elevation [98]. The relationship between BAs and the microbiota underscores FXR's role in liver-intestinal circulation, further demonstrated through its influence on microbiota regulation. Degirolamo et al. demonstrated that probiotics induce microbiota regulation and enhance BA deconjugation, influencing variations in ileal BA assimilation and inhibiting the enterohepatic FXR-FGF15 axis [99]. FXR is also implicated in microbiota metabolism through flavin monooxygenases3, thereby affecting the occurrence of certain metabolic diseases such as atherosclerosis [100]. Consequently, the development of FXR agonists or antagonists targeting the gut flora-BA-gut FXR axis holds potential for treating related metabolic disorders.

**Figure 5.**
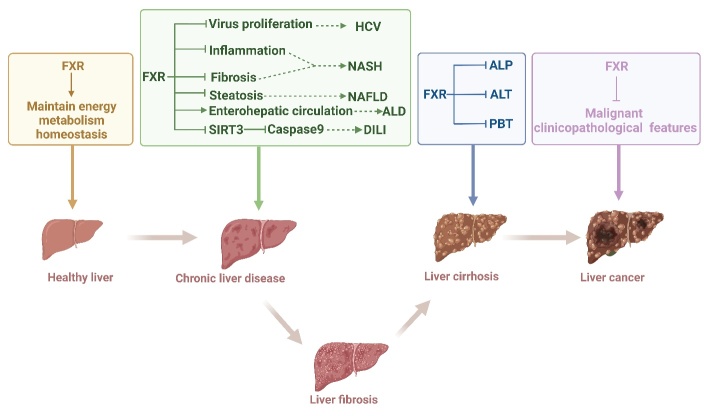
**Role of FXRs in the liver**. FXR activation is used in multiple liver diseases, such as liver injury, fibrosis, and even cancer, and the main mechanism is closely related to inflammation, steatosis, and apoptosis via related factors.

#### 2.2.5. FXR in liver disease

As a pivotal factor in metabolic regulation, FXR plays a crucial role in various liver diseases, including alcoholic liver disease (ALD), NAFLD, viral hepatitis, and drug-induced liver injury (Fig. 5) [101, 102]. Particularly noteworthy is the work of Hartmann and colleagues, who have shown that alcohol leads to alterations in the BA profile due to the reduced activity of FXR [103]. Throughout these processes, FXR upregulates the expression of FGF15 and the BSEP, while concurrently suppressing the expression of CYP8B1 and CYP7A1, all of which collectively contribute to the inhibition of BA synthesis [104]. Furthermore, exposure to alcohol disrupts the interaction between RXRα and FXR, facilitated by the acetylation of FXR, ultimately resulting in the inactivation of FXR [105]. Additionally, the expression of FXR is modulated by proteins whose expressions undergo changes in liver disease, such as sirtuin 1 [66], liver X receptors, PPARs, and others [106, 107]. Much like its role in ALD, studies by Clifford et al. have demonstrated that hepatic FXR controls the expression of genes implicated in adipogenesis, while intestinal FXR regulates lipid absorption [72]. Moreover, FXR agonists, like GSK2324, are employed in the treatment of NAFLD due to their role in decreasing fatty acid absorption and selectively reducing fatty acid synthesis [72]. Additionally, in individuals with HCV infection, FXR participates in lipid oxidation and ketogenesis. Notably, the activation of PPARα and FXR during HCV infection hampers the viral life cycle and potentially forms part of the host's metabolic antiviral response to the infection [108, 109]. Furthermore, antagonism toward FXR might underlie certain occurrences, such as how indomethacin can induce STAT3 phosphorylation, subsequently promoting caspase 9 activation and contributing to drug-induced liver injury [110]. Building upon these insights, our prior research has also delved into the regulatory function of FXR in lipid metabolism induced by alcohol and a high-fat diet. Our findings suggest that traditional Chinese medicine can modulate FXR, thereby ameliorating ALD and NAFLD. Examples of such medicinal agents include *Araliaceae* and *Allium victorialis L* [111, 112]. Beyond the scope of liver injury, FXR also exerts regulatory influence over liver fibrosis. OCA, the pioneering drug targeting FXR, effectively lowers BA levels and impedes the activation of HSCs, thus mitigating fibrosis progression without compromising hepatocyte apoptosis [25]. Significantly, the discovery of OCA dates back to 2002 as 6ECDCA [113]. Initial research unveiled that 6ECDCA, a semisynthetic bile acid derivative, prompts the upregulation of SHP and BSEP, coupled with downregulation of cyp7a1, cyp8b1, and NTCP at the mRNA level [114]. Furthermore, 6-ECDCA enhances insulin-induced differentiation of preadipocytes by modulating the expression of adipocyte-related genes [115], underscoring the potential of exploiting FXR ligands as a promising avenue for addressing cholestatic disorders and fibrosis improvement.

Importantly, the expression of hepatic FXR demonstrates an inverse correlation with various malignant clinical and pathological features of liver cancer. These features encompass outcomes of liver disease, such as tumor size, clinical classification of liver cancer, cancer cell differentiation, and tumor tissue encapsulation [58]. Given the paucity of clinically approved drugs for liver fibrosis, targeting FXR regulation holds promise as a potential breakthrough avenue.

**Figure 6.**
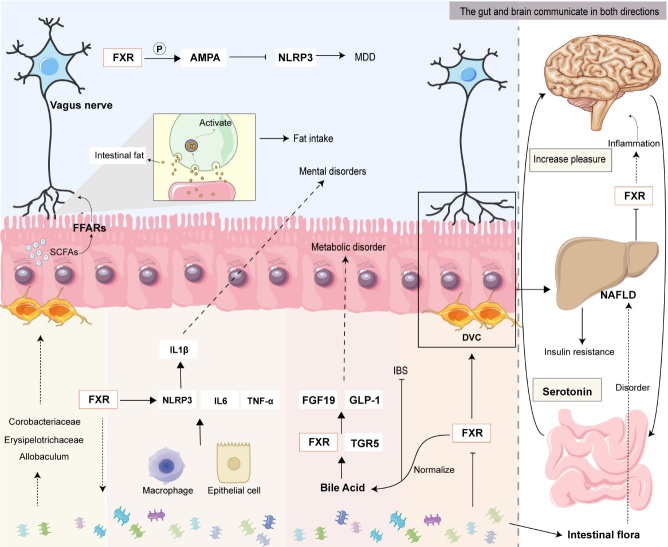
**FXR in the gut-brain axis**. FXRs interact and work together by regulating inflammatory responses, BAs, and the intestinal microbiome involved in entero-brain circulation.

#### 2.2.6. FXR in gut-brain Axis

Apart from its involvement in intestinal-hepatic circulation, the significance of FXR in the intestinal-brain axis should not be underestimated (as illustrated in Fig. 6). The gut-brain axis constitutes a bidirectional communication network connecting the brain and the intestine, encompassing neural pathways, endocrine pathways, immune pathways, and the gut microbiota [116]. Research has highlighted that neuron within the vagus nerve, which connects the intestine and the brain, become activated in response to intestinal fat. These neurons transmit information from the intestine to the brain. In instances where the activity of fat-sensing endothelial cells on the inner wall of the intestine is impeded, signal transmission can be halted, leading to a reduction in mice's appetite for fat [117]. This mechanism has emerged as a significant target for addressing NASH and obesity. Additionally, it has been observed that human mood can affect microbiota composition. Notably, gastrointestinal disorders regulated by intestinal neurons often co-occur with fatty liver disease [118]. This phenomenon arises primarily due to chronic psychological stress inducing shifts in intestinal flora diversity and an increase in intestinal permeability. These changes are accompanied by the release of lipopolysaccharides (LPS) from the intestine into the liver, triggering the activation of the Toll-like receptor 4 (TLR4) signaling pathway, all of which collectively contribute to liver injury [119]. Likewise, alterations in the microbiota can reciprocally influence the body's emotional state [120]. This is due to the gut flora's capacity to synthesize corresponding neurotransmitters that facilitate communication between the gut and the brain. Furthermore, pathogenic microorganisms and bacterial metabolites can stimulate the secretion of proinflammatory cytokines, such as IL-1β, IL-6, and TNF-α. These cytokines not only influence macrophage recruitment within the liver but also directly affect brain function, thereby participating in the emergence of neurological disorders [121]. Of paramount importance is the role of BAs in transmitting signals to the central nervous system through two distinct pathways: a direct central FXR-TGR5 signal and an indirect pathway involving the activation of intestinal FXR and TGR5, leading to the induction of FGF19 and GLP-1 [122-124]. FXR is not confined to the gut and liver; it is also expressed in the brain, indicating its potential circulatory effect. This circulatory effect can be categorized into three key aspects: 1) disruption of the release of proinflammatory cytokines and alleviation of intestinal inflammation; 2) modulation of the body's lipid and energy metabolism through BA regulation; and 3) enhancement of the composition of the gut microbiome. Drawing from these insights, Bao et al. discovered that ganoderic acid A curtailed NLRP3 inflammasome activity and heightened the expression of AMPA receptors in the prefrontal cortex of mice by regulating FXR. As a result, encephalitis activity was suppressed, ultimately ameliorating major depressive disorder [125]. These findings underscore FXR's propensity to interact synergistically with the aforementioned three areas within the gut-brain circulation, thereby assuming a pivotal role in liver fibrosis.

## 3. Pharmacology of FXR in liver fibrosis

In fact, systemic FXR deficiency in mice leads to Indeed, the absence of systemic FXR in mice results in elevated liver BA levels and ensuing liver damage, encompassing hepatic steatosis, inflammation, and fibrosis. The emergence of liver fibrosis is contingent not only upon the activation of HSCs but also on the intimate involvement of hepatocyte injury, immune cell activation, and the modulation of the liver microenvironment, among other factors. Consequently, in the pursuit of preventing and managing liver fibrosis, it becomes imperative to comprehensively grasp the underlying pathogenesis of liver fibrosis and the intricate regulatory role that FXR plays within distinct cell types that contribute to the fibrotic process.

### 3.1 Liver fibrosis: an overview

**Figure 7.**
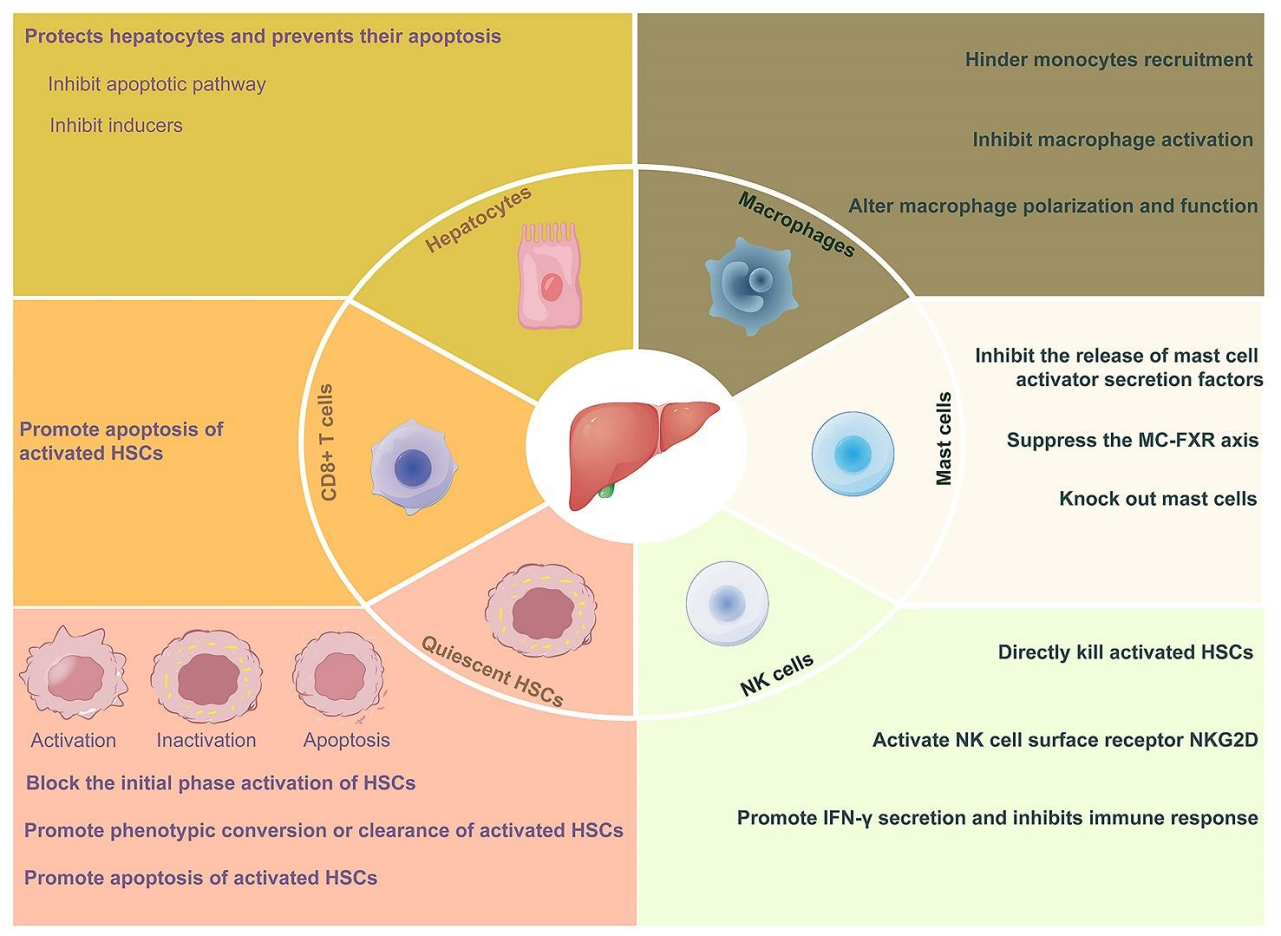
**Anti-fibrosis therapy in liver fibrosis**. According to the pathogenesis, the treatment of liver fibrosis can be started from the cells involved in liver fibrosis. The main methods are inhibiting the damage and apoptosis of liver cells, promoting the type of transformation of immune cells, inhibiting the activation of HSCs, and promoting the apoptosis of activated HSCs.

The development of hepatic fibrosis primarily arises from a sequence of disruptions to liver tissue structure caused by the imbalance between ECM synthesis and degradation [126]. Serving as the initial phase of liver fibrosis, diverse pathogenic factors, such as alcohol, high-fat diets, drugs, and viruses, can induce liver damage. However, when the extent of damage surpasses the liver's inherent repair capacity [127], the liver's elasticity wanes, progressively culminating in liver fibrosis [128]. At this juncture, patients often present with clinical symptoms such as fatigue, appetite loss, altered stool patterns, liver discomfort, and a pallid complexion. If left undiagnosed and untreated, liver fibrosis can advance to cirrhosis and even liver cancer, profoundly impacting quality of life. Clinical investigations have underscored the significance of certain serum markers in relation to the intensity of the inflammatory response and fibrosis. Parameters such as red blood cells, blood platelets, N-terminal procollagen III (PIII NP), and aspartate aminotransferase (AST) exhibit meaningful correlations with the extent of inflammation. Additionally, albumin, the albumin/globulin ratio [129], and PGA and PGAA levels have been linked to inflammation and fibrosis [130]. Notably, hyaluronic acid serves as a precise variable for evaluating inflammation and fibrosis severity in the liver [131]. Hence, the synergistic application of serum markers has the potential to enhance the accuracy of fibrosis diagnosis [132, 133]. With the continual advancement of molecular biology techniques and the pressing need for effective treatments, the intricate mechanisms underpinning liver fibrosis have been gradually unveiled [19]. Broadly, cells exerting pivotal roles in liver fibrosis can be broadly classified into three categories. The first group comprises fibroblasts, including HSCs and epithelial cells, which, upon liver injury, undergo activation and transform into myofibroblasts (MFs) [134, 135]. The second category encompasses signaling cells, such as hepatocytes, hepatic sinus endothelial cells, and bile duct cells, that modulate MF activation and trigger profibrotic pathways [14]. The third class encompasses regulatory cells or immune cells, such as Kupffer cells, natural killer cells, macrophages, and mast cells. These cells can dynamically adapt their roles in response to the onset and regression of liver fibrosis [136, 137]. Throughout chronic liver injury, hepatocytes sustain damage and even undergo apoptosis, releasing damage-related molecular patterns. This triggers a persistent inflammatory milieu characterized by macrophage and immune cell infiltration, accompanied by the release of profibrotic cytokines such as TGF-β, TNF-α, and PDGF. Within this continuum, HSCs become activated and differentiate into MF, identifiable by the expression of α-smooth muscle actin (α-SMA) and collagen I—an ECM component—ultimately driving the onset of liver fibrosis [138-140] (Fig 7). Hence, a comprehensive exploration of FXR's roles within distinct cell types is pivotal for a deeper comprehension of its mechanisms in liver fibrosis.

### 3.2. FXR with liver fibrosis

**Figure 8.**
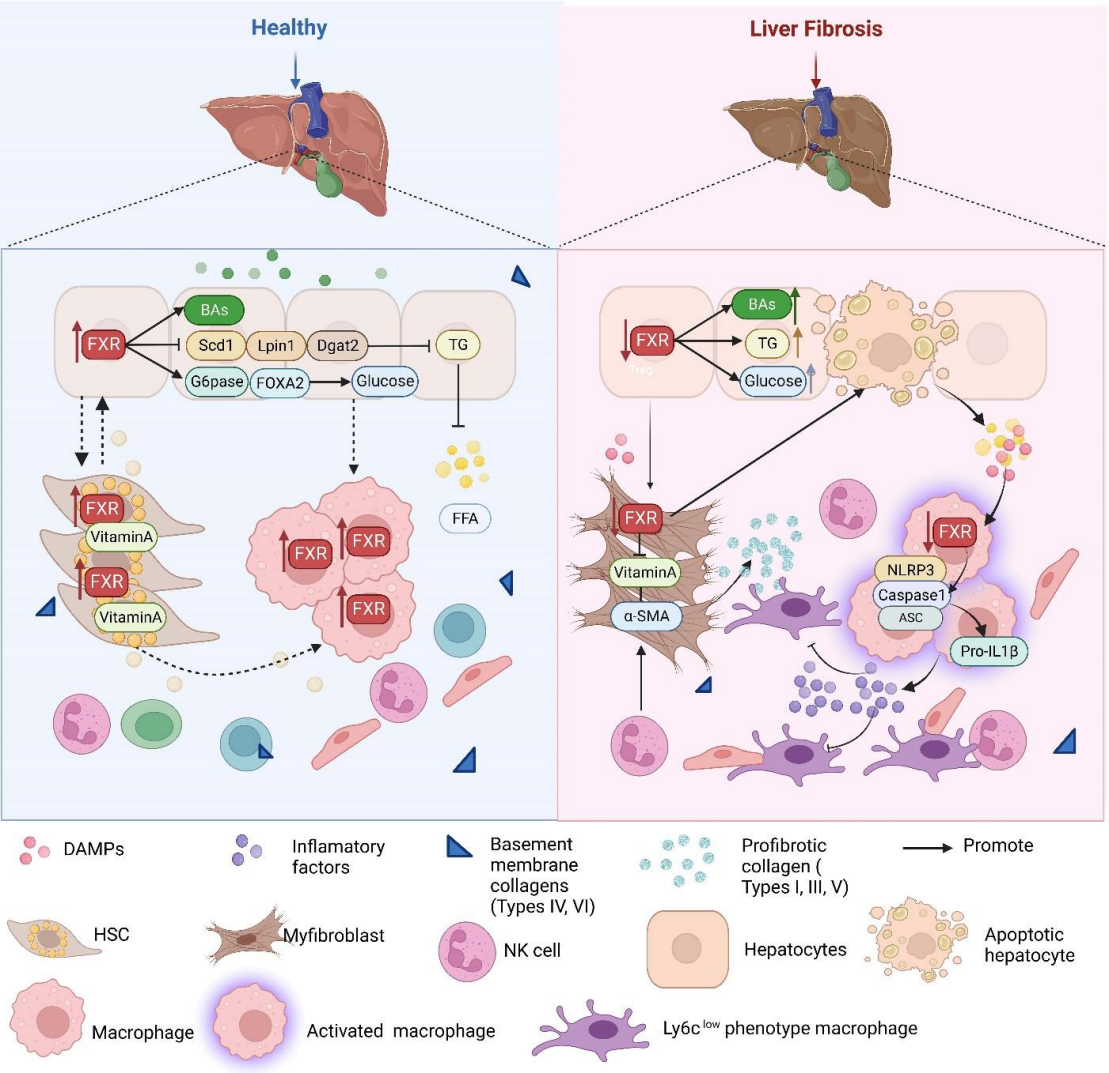
**Summary of the role of FXR in key cells in liver fibrosis**. In the normal liver, hepatocytes provide energy and nutrients to nonparenchymal cells. Vitamin A stored in HSCs promotes the growth and proliferation of hepatocytes and macrophages, effectively maintaining homeostasis in the liver microenvironment. After liver injury, FXR was decreased significantly, accompanied by apoptosis of hepatocytes as well as the release of increased DAMPs and FFA content, resulting in macrophage activation. During this process, HSCs lose the storage function of vitamin A and contribute to the deposition of ECM, leading to liver fibrosis.

Systemic FXR deficiency in mice leads to elevated liver BA levels and subsequent liver disorders, including liver steatosis, inflammation, and fibrosis. The development of liver fibrosis involves not only the activation of HSCs but also close interactions with hepatocyte injury, immune cell activation, and alterations in the liver microenvironment. Currently, the range of therapeutic drugs approved by the FDA remains limited, predominantly due to the constraints of existing methodologies for assessing fibrosis. While ultrasound, elastography, MRI, and CT serve as primary tools for diagnosing liver disease [141], the endorsement and clinical evaluation of novel liver fibrosis drugs necessitate invasive liver biopsies. This approach is susceptible to variations between samples and observers and scaling it up to accommodate the approximately 1 billion affected individuals worldwide proves challenging [142]. In contrast, noninvasive tests using biomarkers assume a pivotal role in fibrosis diagnosis, staging, and continuous monitoring [143]. Presently, widely used noninvasive methods such as the FIB-4 index, and liver stiffness measurement carry a notable risk of false positives and outcome uncertainty [144]. Thus, delving into the distinctive roles of FXRs within various cell types emerges as highly significant for advancing the prevention and treatment of liver fibrosis (Fig. 8).

#### 3.2.1. FXR in hepatocytes

In a healthy liver, hepatocytes constitute approximately 70% of the total cell population and primarily oversee metabolic processes crucial to liver functions, including complement factor regulation, bile acid synthesis, and gluconeogenesis [145]. FXRs, not confined solely to bile acid metabolism, contribute to the reduction of de novo liver fat via the FXR-SHP-SREBP1c pathway, thereby curbing liver triglyceride accumulation [71]. Recent comprehensive lipidomics analysis has revealed that FXR activation diminishes the levels of PUFAs and MUFAs in the liver. This effect hinges on two distinct pathways: inhibition of lipogenesis gene expression and lipid absorption [72]. Additionally, FXRs participate in transcriptional-level regulation of miR-552-3p-controlled metabolic genes in glycolipid metabolic disorders through cis-binding [73]. Concerning the modulation of glucose metabolism, FXRs oversee liver glucose production through the activation of gluconeogenic-related genes and glucagon-triggered FOXA2 [74].

Upon encountering stimuli, hepatocytes undergo changes in their secretion profiles and the gene expression of specific proinflammatory factors. These alterations bear an inverse relationship with FXR expression in the liver [146]. Furthermore, cystathionine γ-lyase (CSE) and its resultant product hydrogen sulfide (H_2_S) generated in hepatocytes, which demonstrate decreased levels in NAFLD mice or hepatocyte models, can trigger posttranslational FXR modification at the Cys138/141 site. This mechanism can function as an FXR activator, modulating glycolipid metabolism and fibrosis [147, 148]. Similarly, FXR can bind to shared sites in the Sox9 promoter to boost glycolysis and ATP production. Consequently, this facilitates the regeneration of Sox9+ hepatocytes that assume the role of bidirectional progenitor cells following liver injury. These cells can give rise to hepatocytes or bile duct cells, contributing to liver repair and regeneration [149, 150]. Furthermore, specific FXR knockout in hepatocytes significantly delays the initiation signal of growth factors, hampering liver regeneration [151].

At present, several drugs targeting FXR have been developed to address various stages of liver cell damage. For instance, EDP-305, an oral synthetic FXR agonist, has demonstrated efficacy in reducing liver fat content and serum ALT levels in phase II trials. This supports the need for extended trials evaluating histological endpoints in NASH [152]. Vonafexor, another FXR agonist, has exhibited the ability to decrease HBV surface antigen levels in CHB within a short timeframe and has demonstrated certain safety parameters. This implies its potential role in preventing hepatocyte injury, although extensive trials are required to assess its therapeutic viability [153]. Nevertheless, Wang et al. highlighted that FXR overexpression, rather than its ligands, enhances its interaction with CASP8, thereby suppressing hepatocyte apoptosis in hepatic fibrosis [12]. Consequently, gaining a comprehensive understanding of FXR's roles within hepatocytes is pivotal for the development of FXR-targeting drugs and the treatment of liver fibrosis.

#### 3.2.2. FXR in immune cells

Positioned at the junction of the immune system and antigens from the gastrointestinal tract, the liver is primarily endowed with innate immunocytes [154-156], which encompass Kupffer cells (a subtype of macrophages) [157, 158], natural killer (NK) cells, neutrophils, NK T (NKT) cells, monocytes, and dendritic cells [159]. This attribute designates the liver as an immune organ [160]. Research has illuminated that FXR's scope extends beyond hepatocyte metabolism and injury; it also influences immune cell activation. Specifically, FXR is expressed within immune cells and exerts regulatory effects on them [161]. For instance, the expression of FXR in KCs surpasses that in bone marrow-derived macrophages. Pretreatment of KCs with GW4064, rather than bone marrow-derived macrophages, inhibits TNF-α and elevates IL-10 levels upon TLR stimulation. Notably, SHP, an intrinsic negative factor in TLR-triggered inflammatory responses, plays a pivotal role in FXR's immune regulation in KCs [162]. Broadly speaking, FXR activation in KCs can dampen proinflammatory responses in the liver through heightened SHP expression [163]. Additionally, activated FXR fosters the generation of anti-inflammatory macrophages, enhances IL-10 secretion, and curbs T-cell responses. This phenomenon has been validated in human monocytes from both healthy controls and multiple sclerosis patients [164]. Furthermore, FXR physically interacts with NLRP3 and caspase 1, thereby exerting a negative regulatory influence on NLRP3 inflammasome activation in macrophages [165]. Notably, recent clinical trials utilizing OCA for NASH therapy have indicated a potential modulation of the NLRP3 inflammasome, hinting at the potential immunological mechanisms underlying OCA's effects [166, 167]. Moreover, the transformation of macrophages into an anti-inflammatory phenotype is intertwined with the production and elimination of osteopontin by NKT cells [168]. Activated FXR, in turn, can inhibit osteopontin production by engaging with its promoter in NKT cells through SHP [169]. These findings collectively underscore FXR's role in modulating NKT cell activation within the liver. Additionally, bile acid metabolism affects the aggregation of NKT cells by regulating the expression of CXCL16 in hepatic sinusoidal endothelial cells. This selective role plays a part in tumor suppression, a notion that has recently been corroborated by human HCC single-cell RNA-seq data. Building upon this foundation, Gou et al. observed that OCA combined with a TGR5 antagonist demonstrates certain antitumor activity [171]. This finding provides groundwork and potential strategy for NKT cell-based immunotherapy for HCC. Somewhat distinct from NKT cells, clinical investigations have unveiled that the number of mast cells and the expression of FXR in the biliary tract are elevated in patients with liver injury compared to control subjects. In this context, mast cells can regulate intestinal inflammation and bile duct responses triggered by cholestasis via the FXR signaling pathway [172]. While preclinical FXR activation trials deliver negative regulatory signals that mitigate immune dysfunction in various liver diseases driven by inflammation, the clinical implications of these mechanisms in FXR-related conditions remain uncertain [173]. This underscores the ongoing endeavor to explore the clinical modulation of FXR and its agonists on immune cells.

#### 3.2.3. FXR in HSCs

HSCs, constituting 15% of the hepatocyte population, reside in the Disse cavity in close proximity to sinusoidal endothelial cells and hepatocytes. These cells, primarily acting as fibroblasts, play a pivotal role in the progression of hepatic fibrosis [146]. Under normal conditions, HSCs are typically quiescent, primarily serving as reservoirs for storing vitamin A [174]. Notably, vitamin A directly influences bile acid synthesis and transport through nuclear receptors, including FXR [175]. Similarly, FXR-deficient mice exhibit reduced vitamin A storage capacity, a phenomenon ameliorated upon FXR reintroduction into the hepatic system [176]. As early as 2004, Fiorucci et al. reported that FXR is also present in HSCs and serves as a negative regulatory factor, extending its influence beyond bile acid metabolism [177]. FXR expression in primary HSCs and the HSC-T6 rat immortalized cell line [178] effectively inhibits collagen I mRNA expression and collagen synthesis [177]. Nevertheless, FXR activation does not hinder HSC proliferation or their transition into myofibroblast-like states, seemingly in contrast with FXR's purported antifibrotic effects. In reality, FXR exerts its influence by modulating the transcription of target genes, notably SHP [179, 180]. Studies have demonstrated that FXR ligands upregulate SHP expression at the mRNA level in HSCs, analogous to hepatocytes [181]. Importantly, the FXR-SHP-mediated regulatory cascade contributes to collagen remodeling induced by FXR ligands. Interestingly, manipulating SHP expression, whether through silencing or overexpression, nullifies the regulatory impact of FXR ligands on the expression of 1(I) collagen at the mRNA level, all of which showed that the. These observations underscore the SHP-dependent nature of FXR's regulatory mechanism in HSCs. Concurrently, FXR knockdown leads to an increase in tissue inhibitor of metalloproteinase 1 (TIMP1), a key participant in ECM formation within HSCs [182]. Upon activation by inflammatory mediators, such as TGF-β, quiescent HSCs transition to an activated state, participating in ECM deposition [183]. In this context, a selective FXR agonist (BAR704) can obstruct TGF-β promoter binding to p-SMAD3 through SHP-mediated transcription, consequently impeding HSC transdifferentiation and attenuating liver fibrosis [184]. Furthermore, activated FXR can influence HSC contraction through either sphingosine-1-phosphate receptor 2-mediated Ca^2+^ sensitization or Ca^2+^ dependent mechanisms [185]. Drawing from these insights, the exploration of FXR-targeting drugs for inhibiting HSC activation presents a promising avenue in the treatment of liver fibrosis.

#### 3.2.4. FXR in enterohepatic circulation

In addition to primary liver cells, liver fibrosis is intricately linked to hepatoenteric circulation [186]. This connection predominantly arises from bacterial and microbiome derivatives, bile acids, physiological function, and intestinal barrier integrity [187]. For instance, in children with NAFLD, a decline in biodiversity and significant enrichment of genes involved in LPS synthesis were observed. This phenomenon might contribute to increased intestinal permeability and the promotion of a proinflammatory state, which is positively associated with liver fibrosis [188-190]. LPS-induced dysregulation of intestinal immunity and the microbiome is intertwined with changes in bile acid metabolism [191]. As a receptor for LPS, TLR4 is inhibited by activated FXR, regulating the expression of proteins linked to the TLR4/NF-κB signaling pathway, such as MyD88, p-p65, and p-IκBα. This mechanism shields the liver from enterogenic hepatitis [192]. Furthermore, research by Jiang et al. highlighted a significant reduction in the abundance of intestinal Prevotella copri in primary sclerosing cholangitis. However, treatment with Prevotella copri was shown to enhance hepatic fibrosis by activating the FXR pathway [193]. Similarly, intestinal Prevotella copri was found to restore glucose homeostasis by enhancing bile acid metabolism and FXR expression [194]. Additionally, the degradation of tight junction and adherens junction proteins in the intestinal tract accelerates apoptosis and nitrification of enterocytes. This process contributes to ALD-induced intestinal leakage and endotoxemia, triggering an inflammatory response in the liver [195]. Subsequent studies identified that occludin deficiency heightened the sensitivity of mice to ethanol-induced liver damage [196]. However, activated FXR can counteract intestinal epithelial barrier injury induced by LPS through the regulation of transepithelial resistance and tight junction proteins [197]. Central to this context is the hepatoenteric circulation, which governs the uptake of bile acids and other steroids metabolized or produced in the liver. These compounds are then secreted into the enteric canal, with a subsequent reabsorption back into circulation before returning to the liver. This process underscores the crucial role of FXR in hepatoenteric circulation [95]. Presently, the probiotic Lactobacillus rhamnosus GG has been shown to reduce the liver concentration of the FXR antagonist T-βMCA and increase the FXR agonist chenodeoxycholic acid. This results in elevated serum and ileal FGF15 levels, ultimately ameliorating BDL-induced hepatic fibrosis in mice [198]. Moreover, the FXR novel antagonist glycine-β-muricholic acid, which is retained in the gut, has the potential to enhance glycolipid metabolism in obesity and NAFLD [199]. Consequently, FXR agonists or inhibitors targeting enterohepatic circulation might hold promise for therapeutic interventions in hepatic fibrosis.

#### 3.2.5 Exploration of FXR-related pathways and mechanisms in liver fibrosis

As previously highlighted, while FXR serves as a pivotal target, it seldom operates in isolation within disease contexts, often entwined with other pathways. When considering the induction of liver fibrosis formation, FXR-related pathways can be classified into various facets, the foremost being its involvement in fat synthesis. FXR plays a role in modulating lipid homeostasis through its influence on de novo lipogenesis, HDL formation, hepatic uptake of HDL, and β-oxidation of fatty acids. This regulatory impact stems from its control over the expression of key players, including SREBP1, PLTP, SCARB1, and PPARα [200-202]. Regarding inflammation, FXR activation exhibits the ability to curtail the production of proinflammatory cytokines, such as IL1β and NOS_2_. This effect primarily stems from the trans-repression of TLR4 and the modulation of NLRP3 inflammasome assembly [203]. Moreover, oxidative stress and other contributing factors further underscore FXR's connections with AMPK, SIRT6, and NRF2 [204-206]. Hence, delving into the intricate interactions between FXR and other pathways holds paramount importance in surmounting the therapeutic challenges surrounding FXR and devising more effective treatments for liver fibrosis.

#### 3.2.6. Interactions between FXR and other molecular targets

As a nuclear receptor, RXR typically forms heterodimers with other nuclear receptors, such as FXR/RXR, LXR/RXR, and PPAR/RXR, thereby orchestrating the expression of downstream target genes [207]. This underscores FXR's capacity to interact with other receptors or signaling pathways within physiological contexts. Our previous research revealed that FXR knockdown leads to a downregulation of PPARα and LXRα expression, subsequently fostering SREBP1 expression and lipid accumulation [112]. In the realm of maintaining liver energy metabolism homeostasis, PPARα and FXR work synergistically to curb lipid production [208]. Therapies targeting PPARα and FXR agonists have been employed for the treatment of NAFLD [209]. Diverging slightly from the role of PPARα, FXR and PPARγ exert counterregulatory functions on HSCs, thereby hindering the progression of liver fibrosis in rodent models. To elucidate, transdifferentiation of HSCs involves the inhibition of PPARγ mRNA expression, while FXR ligand treatment augments PPARγ expression and suppresses type I collagen accumulation induced by TGFβ, amplifying the antifibrotic potential of PPARγ [210]. Furthermore, the heterodimer formed by FXR and RXR binding enhances FXR's ligand-binding capability, with this effect being linked to the augmentation of RXRα ligands [211]. Correspondingly, Jenniskens et al. demonstrated that FXR/RXR heterodimers effectively curtail bile acid synthetic feedback, thus mitigating intrahepatic cholestasis and ameliorating liver fibrosis-related symptoms [212]. Additionally, chenodeoxycholic acid can bolster GLP-1 production and secretion in enteral endocrine cells by simultaneously activating TGR5 and inhibiting FXRs. This interplay leads to improved glucose homeostasis, indicating a prospective anti-hepatic fibrosis effect [213]. Notably, FXR and TGR5 not only harmonize in regulating bile acids but also contribute to microbiota regulation [172]. Consequently, the FXR/TGR5 dual receptor agonist INT-767 has been employed for conditions linked to glucose-lipid metabolism imbalances [214]. While the synergistic interplay between FXR and other factors or ligands holds promise for metabolic disease treatment, it simultaneously poses challenges in developing highly selective FXR modulators.

## 4. Clinical significance of FXR regulators in liver fibrosis

Currently, several FXR regulators are being investigated for the treatment of chronic liver diseases in clinical research stages, offering valuable insights for clinical approaches to liver fibrosis treatment. For instance, OCA, an FXR agonist developed by Intercept, shows promise in treating various liver conditions and may enhance survival rates [24]. Specifically, a phase 3 randomized, double-blind, multicenter, placebo-controlled international trial focused on NASH without cirrhosis patients revealed that OCA significantly alleviates fibrosis in NASH patients [215]. However, patients treated with OCA experienced pruritus and impaired quality of life [215, 216]. Furthermore, a randomized controlled trial demonstrated that OCA treatment during liver disease management led to elevated cholesterol levels, correlated with an increase in small and large LDL particles, as well as a decrease in HDL particles at week 12, which improved after discontinuation of the drug for 24 weeks [216]. In response, Pockros et al. proposed combining OCA and atorvastatin. In this approach, patients took atorvastatin (10 mg/day) after four weeks of OCA administration. The results showed that atorvastatin reduced LDLc and LDLpc levels induced by OCA by week 8, indicating that this combination is well tolerated and generally safe [217]. Similarly, EDP-305, an oral synthetic FXR agonist, underwent double-blind phase II research on fibrotic NASH patients (without cirrhosis). Patients were randomly assigned to receive EDP-305 or placebo for 12 weeks. The findings demonstrated that EDP-305 decreases liver fat content and serum ALT levels, supporting the need for longer trials assessing histological endpoints in NASH. Notably, adverse events, including pruritus, vomiting, nausea, diarrhea, dizziness, and headache, were reported by patients (≥5%) [152].

Other FXR agonists in phase II clinical trials include tropifexor, vonafexor, and cilofexor. Initially, the safety, tolerability, and pharmacokinetics of tropifexor were evaluated in healthy volunteers, indicating its acceptability in terms of safety and tolerability, with minimal pruritus and transient increases in serum ALT [218]. Subsequent studies by Camilleri et al. found that a once-daily dose of 60 µg tropifexor was well tolerated and safe [219]. However, recent clinical data evaluating the efficacy and safety of tropifexor in NASH patients over 48 weeks revealed that tropifexor could inhibit ALT and HFF up to week 48, but it did not improve AST at week 12. Additionally, patients randomized to receive tropifexor frequently reported dose-related pruritus, aligning with observations from previous FXR agonist trials [220]. Compared to tropifexor monotherapy, the safety profile of tropifexor combined with CVC demonstrated a similar trend, with no new safety signals or deaths and notably lower rates of adverse events such as itching, nausea, and fatigue [221]. It is important to note that this study did not display a synergistic effect on ALT, weight, or histological endpoints, but the possibility of such an effect in other combinations should not be ruled out.

Similarly, Vonafexor led to decreased HBV surface antigen levels in CHB within a few weeks of administration and demonstrated certain safety [153]. Recent research also found that vonafexor was safe and had the potential to suppress liver fat production, improve liver enzymes, and inhibit weight loss while causing a certain percentage of generalized pruritus [222]. Meanwhile, although cilofexor showed little significant difference in its effects compared to placebo on NASH-induced advanced fibrosis (F3-F4) patients, the proportion of patients experiencing pruritus induced by cilofexor was higher than that of placebo. However, combining cilofexor with firsocostat effectively alleviated steatosis and inflammation and lowered ALT, AST, and TBIL, thus improving the fibrosis score (≤F2) [223]. The latest study revealed that the FXR agonist HPG1860 displayed promising antifibrotic effects in clinical treatment for NASH, with reduced pruritus, and the notable finding was that there was no significant elevation of LDL-C within 16 weeks [224]. Other FXR regulators undergoing clinical trials, as listed on ClinicalTrials.gov (https://clinicaltrials.gov/), are outlined in Table 2.

Hence, combining drugs and restructuring FXR agonists may provide avenues to overcome challenges in translating FXR research into clinical practice. However, the development of clinical FXR agonists encompasses various etiologies of liver fibrosis, necessitating consideration of both their efficacy and side effects [130].

**Table 2:** Summary of partial FXR ligands entering clinical trials in liver disease.

Type	Ingredients	Study	Conditions	Status
**Agonists**	OCA	Phase 2 Study on Lipoprotein Metabolism	Primary Biliary Cirrhosis	Completed
**Agonists**	OCA	Evaluation of Pharmacokinetics and Safety	Liver Cirrhosis	Terminated
**Agonists**	Tropifexor (LJN452)	Safety and Tolerability.	Liver Disease	Completed
**Agonists**	Tropifexor (LJN452)	Study of Safety, Tolerability, and Efficacy of a Combination Treatment of LJN452 and CVC	NASH and liver fibrosis	Completed
**Agonists**	Tropifexor (LJN452)	Evaluation of the Pharmacokinetics	NAFLD	Completed
**Agonists**	EYP001a	Food Effect Study in Subjects	Hepatitis B, Chronic	Completed
**Agonists**	EYP001a	Safety, Tolerability, Pharmacokinetics and Pharmacodynamics	NASH	Completed
**Agonists**	ASC41	Evaluate the Safety, Tolerability, and Efficacy	NAFLD and NASH	Recruiting
**Agonists**	ASC41	Evaluate the Safety and Efficacy	Obesity and NAFLD	Completed
**Agonists**	EYP001a	Safety, Tolerability, Pharmacokinetics and Pharmacodynamics	NASH	Completed
**Agonists**	EYP001a	Evaluation of the Safety and Pharmacology	Hepatitis B, Chronic	Completed
**Agonists**	Px-104	Safety Pilot Study	NAFLD	Completed
**Agonists**	TQA3526	Study in the Treatment	Primary Biliary Cirrhosis	Unknown
**Agonists**	MET409	Study to Evaluate Alone or Combination	NASH	Active, not recruiting
**Agonists**	TERN-501	Evaluate the Safety, Efficacy, Pharmacokinetics and Pharmacodynamics	NASH	Recruiting
**Agonists**	TERN-101	Safety, Tolerability, Efficacy, and Pharmacokinetics Study	NASH	Completed
**Agonists**	BAR502	Safety, Tolerability, Pharmacokinetics, and Pharmacodynamics	NAFLD	Not yet recruiting
**Antagonist**	Guggulsterone	Role in Hepatitis C Virus Replication	Chronic Hepatitis C	Terminated

## 5. Concluding Remarks and Future Perspectives

While targeting FXR for the treatment of liver fibrosis encompasses various aspects, its application requires careful selection due to potential concerns. Doubts still exist concerning the research on FXR ligand drugs. For instance, certain studies have questioned whether the timing of FXR agonist administration aligns with the clinical context, yielding less than satisfactory results [152]. Notably, FXR agonist administration is in part aimed at preventing the onset of liver fibrosis. However, findings from clinical trials indicate that liver fibrosis symptoms may have already manifested at the time of treatment initiation, necessitating attention [225]. The efficacy of FXR in regulating hepatic fibrosis is acknowledged, but its underlying mechanism warrants further exploration and refinement.

Furthermore, the potential side effects stemming from FXR activation raise concerns. Pruritus and dyslipidemia are recognized side effects that cannot be entirely circumvented. Current research endeavors seek to mitigate these issues by reducing dosages for symptomatic relief and exploring combinations with other drugs such as statins, CCR2 inhibitors, and ACC inhibitors to mitigate side effects [226]. Despite pruritus-related challenges, OCA has gained FDA approval as a treatment for NASH, with side effects managed through statin use and pruritus-alleviating medications [227]. Presently, the development of clinical drugs targeting FXR ligands primarily focuses on continuous structural optimization, aiming to alleviate side effects, akin to searching for a needle in a haystack. Encouragingly, there have been promising outcomes. Harrison et al. identified MET409, a structurally optimized compound, in clinical trials. MET409 reduces fat content in NASH patients with mild side effects, offering hope in terms of structural optimization of FXR regulators [228].

Although the study of side effects progresses at a relatively gradual pace, it is understandable due to the inherent difficulty in finding suitable models that accurately mimic human responses to side effects [229]. Another reason behind FXR's significance lies in its multifaceted potential for therapeutic applications, which could extend beyond liver fibrosis treatment.

## References

[1] Zhu Y, Liu H, Zhang M, Guo GL (2016). Fatty liver diseases, bile acids, and FXR. Acta Pharm Sin B, 6:409-412. 27709009 10.1016/j.apsb.2016.07.008 PMC5045552

[2] Yan N, Yan T, Xia Y, Hao H, Wang G, Gonzalez FJ (2021). The pathophysiological function of non-gastrointestinal farnesoid X receptor. Pharmacol Ther, 226:107867. 33895191 10.1016/j.pharmthera.2021.107867

[3] Fang Y, Hegazy L, Finck BN, Elgendy B (2021). Recent Advances in the Medicinal Chemistry of Farnesoid X Receptor. J Med Chem, 64:17545-17571. 34889100 10.1021/acs.jmedchem.1c01017

[4] Otte K, Kranz H, Kober I, Thompson P, Hoefer M, Haubold B, et al. (2003). Identification of farnesoid X receptor beta as a novel mammalian nuclear receptor sensing lanosterol. Mol Cell Biol, 23:864-872. 12529392 10.1128/MCB.23.3.864-872.2003 PMC140718

[5] Anakk S, Dean AE (2020). Fxr-alpha Skips Alternatively in Liver Metabolism. Gastroenterology, 159:1655-1657. 32926939 10.1053/j.gastro.2020.09.008

[6] Konigshofer P, Brusilovskaya K, Petrenko O, Hofer BS, Schwabl P, Trauner M, et al. (2021). Nuclear receptors in liver fibrosis. Biochim Biophys Acta Mol Basis Dis, 1867:166235. 34339839 10.1016/j.bbadis.2021.166235

[7] Mencarelli A, Fiorucci S (2010). FXR an emerging therapeutic target for the treatment of atherosclerosis. J Cell Mol Med, 14:79-92. 20041971 10.1111/j.1582-4934.2009.00997.x PMC3837604

[8] De Magalhaes Filho CD, Downes M, Evans R (2016). Bile Acid Analog Intercepts Liver Fibrosis. Cell, 166:789. 27518554 10.1016/j.cell.2016.08.001

[9] Massafra V, van Mil SWC (2018). Farnesoid X receptor: A "homeostat" for hepatic nutrient metabolism. Biochim Biophys Acta Mol Basis Dis, 1864:45-59. 28986309 10.1016/j.bbadis.2017.10.003

[10] Jansen PL, Schaap FG (2014). How sweet it is to activate FXR. Hepatology, 59:1665-1667. 24123073 10.1002/hep.26778

[11] Lee CG, Kim YW, Kim EH, Meng Z, Huang W, Hwang SJ, et al. (2012). Farnesoid X receptor protects hepatocytes from injury by repressing miR-199a-3p, which increases levels of LKB1. Gastroenterology, 142:1206-1217 e1207. 22265968 10.1053/j.gastro.2012.01.007 PMC3578415

[12] Wang H, Ge C, Zhou J, Guo Y, Cui S, Huang N, et al. (2018). Noncanonical farnesoid X receptor signaling inhibits apoptosis and impedes liver fibrosis. EBioMedicine, 37:322-333. 30337250 10.1016/j.ebiom.2018.10.028 PMC6286639

[13] Han X, Wu Y, Yang Q, Cao G (2021). Peroxisome proliferator-activated receptors in the pathogenesis and therapies of liver fibrosis. Pharmacol Ther, 222:107791. 33321113 10.1016/j.pharmthera.2020.107791

[14] Ruart M, Chavarria L, Camprecios G, Suarez-Herrera N, Montironi C, Guixe-Muntet S, et al. (2019). Impaired endothelial autophagy promotes liver fibrosis by aggravating the oxidative stress response during acute liver injury. J Hepatol, 70:458-469. 30367898 10.1016/j.jhep.2018.10.015 PMC6704477

[15] Parola M, Pinzani M (2019). Liver fibrosis: Pathophysiology, pathogenetic targets and clinical issues. Mol Aspects Med, 65:37-55. 30213667 10.1016/j.mam.2018.09.002

[16] Hernandez-Gea V, Friedman SL (2011). Pathogenesis of liver fibrosis. Annu Rev Pathol, 6:425-456. 21073339 10.1146/annurev-pathol-011110-130246

[17] Higashi T, Friedman SL, Hoshida Y (2017). Hepatic stellate cells as key target in liver fibrosis. Adv Drug Deliv Rev, 121:27-42. 28506744 10.1016/j.addr.2017.05.007 PMC5682243

[18] Sun Y, Zhou J, Wang L, Wu X, Chen Y, Piao H, et al. (2017). New classification of liver biopsy assessment for fibrosis in chronic hepatitis B patients before and after treatment. Hepatology, 65:1438-1450. 28027574 10.1002/hep.29009

[19] Bataller R, Brenner DA (2005). Liver fibrosis. Journal of Clinical Investigation, 115:209-218. 15690074 10.1172/JCI24282 PMC546435

[20] Cowan ML, Rahman TM, Krishna S (2010). Proteomic approaches in the search for biomarkers of liver fibrosis. Trends Mol Med, 16:171-183. 20304704 10.1016/j.molmed.2010.01.006

[21] Sung H, Ferlay J, Siegel RL, Laversanne M, Soerjomataram I, Jemal A, et al. (2021). Global Cancer Statistics 2020: GLOBOCAN Estimates of Incidence and Mortality Worldwide for 36 Cancers in 185 Countries. CA Cancer J Clin, 71:209-249. 33538338 10.3322/caac.21660

[22] Myojin Y, Hikita H, Sugiyama M, Sasaki Y, Fukumoto K, Sakane S, et al. (2021). Hepatic Stellate Cells in Hepatocellular Carcinoma Promote Tumor Growth Via Growth Differentiation Factor 15 Production. Gastroenterology, 160:1741-1754.e1716. 33346004 10.1053/j.gastro.2020.12.015

[23] Han CY, Rho HS, Kim A, Kim TH, Jang K, Jun DW, et al. (2018). FXR Inhibits Endoplasmic Reticulum Stress-Induced NLRP3 Inflammasome in Hepatocytes and Ameliorates Liver Injury. Cell Rep, 24:2985-2999. 30208322 10.1016/j.celrep.2018.07.068

[24] Markham A, Keam SJ (2016). Obeticholic Acid: First Global Approval. Drugs, 76:1221-1226. 27406083 10.1007/s40265-016-0616-x

[25] Zhou J, Huang N, Guo Y, Cui S, Ge C, He Q, et al. (2019). Combined obeticholic acid and apoptosis inhibitor treatment alleviates liver fibrosis. Acta Pharm Sin B, 9:526-536. 31193776 10.1016/j.apsb.2018.11.004 PMC6542786

[26] Trauner M, Fuchs CD (2022). Novel therapeutic targets for cholestatic and fatty liver disease. Gut, 71:194-209. 34615727 10.1136/gutjnl-2021-324305 PMC8666813

[27] Downes M, Verdecia MA, Roecker AJ, Hughes R, Hogenesch JB, Kast-Woelbern HR, et al. (2003). A chemical, genetic, and structural analysis of the nuclear bile acid receptor FXR. Mol Cell, 11:1079-92. 12718892 10.1016/s1097-2765(03)00104-7 PMC6179153

[28] Han CY, Rho HS, Kim A, Kim TH, Jang K, Jun DW, et al. (2018). FXR Inhibits Endoplasmic Reticulum Stress-Induced NLRP3 Inflammasome in Hepatocytes and Ameliorates Liver Injury. Cell Reports, 24:2985-2999. 30208322 10.1016/j.celrep.2018.07.068

[29] Wisely GB, Miller AB, Davis RG, Thornquest AD, Johnson R, Spitzer T, et al. (2002). Hepatocyte nuclear factor 4 is a transcription factor that constitutively binds fatty acids. Structure, 10:1225-1234. 12220494 10.1016/s0969-2126(02)00829-8

[30] Sladek F (2002). Desperately seeking..something. Molecular Cell, 10:219-221. 12191466 10.1016/s1097-2765(02)00605-6

[31] Lee FY, Lee H, Hubbert ML, Edwards PA, Zhang Y (2006). FXR, a multipurpose nuclear receptor. Trends Biochem Sci, 31:572-580. 16908160 10.1016/j.tibs.2006.08.002

[32] Schierle S, Neumann S, Heitel P, Willems S, Kaiser A, Pollinger J, et al. (2020). Design and Structural Optimization of Dual FXR/PPARdelta Activators. J Med Chem, 63:8369-8379. 32687365 10.1021/acs.jmedchem.0c00618

[33] Merk D, Sreeramulu S, Kudlinzki D, Saxena K, Linhard V, Gande SL, et al. (2019). Molecular tuning of farnesoid X receptor partial agonism. Nat Commun, 10:2915. 31266946 10.1038/s41467-019-10853-2 PMC6606567

[34] Jiang L, Zhang H, Xiao D, Wei H, Chen Y (2021). Farnesoid X receptor (FXR): Structures and ligands. Computational and Structural Biotechnology Journal, 19:2148-2159. 33995909 10.1016/j.csbj.2021.04.029 PMC8091178

[35] Funabashi M, Grove TL, Wang M, Varma Y, McFadden ME, Brown LC, et al. (2020). A metabolic pathway for bile acid dehydroxylation by the gut microbiome. Nature, 582:566-570. 32555455 10.1038/s41586-020-2396-4 PMC7319900

[36] Sonne DP, van Nierop FS, Kulik W, Soeters MR, Vilsbøll T, Knop FK (2016). Postprandial Plasma Concentrations of Individual Bile Acids and FGF-19 in Patients With Type 2 Diabetes. J Clin Endocrinol Metab, 101:3002-3009. 27270475 10.1210/jc.2016-1607

[37] Sepe V, Renga B, Festa C, D’Amore C, Masullo D, Cipriani S, et al. (2014). Modification on Ursodeoxycholic Acid (UDCA) Scaffold. Discovery of Bile Acid Derivatives As Selective Agonists of Cell-Surface G-Protein Coupled Bile Acid Receptor 1 (GP-BAR1). Journal of Medicinal Chemistry, 57:7687-7701. 25162837 10.1021/jm500889f

[38] Jiao N, Baker SS, Chapa-Rodriguez A, Liu W, Nugent CA, Tsompana M, et al. (2018). Suppressed hepatic bile acid signalling despite elevated production of primary and secondary bile acids in NAFLD. Gut, 67:1881-1891. 28774887 10.1136/gutjnl-2017-314307

[39] Fu T, Coulter S, Yoshihara E, Oh TG, Fang S, Cayabyab F, et al. (2019). FXR Regulates Intestinal Cancer Stem Cell Proliferation. Cell, 176:1098-1112 e1018. 30794774 10.1016/j.cell.2019.01.036 PMC6701863

[40] Yang YL, Zhou WW, Wu S, Tang WL, Wang ZW, Zhou ZY, et al. (2021). Intestinal Flora is a Key Factor in Insulin Resistance and Contributes to the Development of Polycystic Ovary Syndrome. Endocrinology, 162. 34145455 10.1210/endocr/bqab118 PMC8375444

[41] Lumbreras S, Ricobaraza A, Baila-Rueda L, Gonzalez-Aparicio M, Mora-Jimenez L, Uriarte I, et al. (2021). Gene supplementation of CYP27A1 in the liver restores bile acid metabolism in a mouse model of cerebrotendinous xanthomatosis. Mol Ther Methods Clin Dev, 22:210-221. 34485606 10.1016/j.omtm.2021.07.002 PMC8399082

[42] Sun L, Xie C, Wang G, Wu Y, Wu Q, Wang X, et al. (2018). Gut microbiota and intestinal FXR mediate the clinical benefits of metformin. Nat Med, 24:1919-1929. 30397356 10.1038/s41591-018-0222-4 PMC6479226

[43] Therdtatha P, Song Y, Tanaka M, Mariyatun M, Almunifah M, Manurung NEP, et al. (2021). Gut Microbiome of Indonesian Adults Associated with Obesity and Type 2 Diabetes: A Cross-Sectional Study in an Asian City, Yogyakarta. Microorganisms, 9. 33922321 10.3390/microorganisms9050897 PMC8147061

[44] Yu J, Lo JL, Huang L, Zhao A, Metzger E, Adams A, et al. (2002). Lithocholic acid decreases expression of bile salt export pump through farnesoid X receptor antagonist activity. J Biol Chem, 277:31441-31447. 12052824 10.1074/jbc.M200474200

[45] Song MH, Shim WS (2022). Lithocholic Acid Activates Mas-Related G Protein-Coupled Receptors, Contributing to Itch in Mice. Biomol Ther (Seoul), 30:38-47. 34263729 10.4062/biomolther.2021.059 PMC8724838

[46] Rinella ME, Dufour JF, Anstee QM, Goodman Z, Younossi Z, Harrison SA, et al. (2022). Non-invasive evaluation of response to obeticholic acid in patients with NASH: Results from the REGENERATE study. J Hepatol, 76:536-548. 34793868 10.1016/j.jhep.2021.10.029

[47] Salhab A, Amer J, Lu Y, Safadi R (2022). Sodium(+)/ taurocholate cotransporting polypeptide as target therapy for liver fibrosis. Gut, 71:1373-1385. 34266968 10.1136/gutjnl-2020-323345 PMC9185811

[48] Shim S, Krishnaiah M, Sankham MR, Kim I, Lee Y, Shin I, et al. (2022). Discovery of (E)-3-(3-((2-Cyano-4'-dimethylaminobiphenyl-4-ylmethyl)cyclohexanecarbonylamino)- 5-fluorophenyl)acrylic Acid Methyl Ester, an Intestine-Specific, FXR Partial Agonist for the Treatment of Nonalcoholic Steatohepatitis. J Med Chem, 65:9974-10000. 35797110 10.1021/acs.jmedchem.2c00641

[49] Patel K, Harrison SA, Elkhashab M, Trotter JF, Herring R, Rojter SE, et al. (2020). Cilofexor, a Nonsteroidal FXR Agonist, in Patients With Noncirrhotic NASH: A Phase 2 Randomized Controlled Trial. Hepatology, 72:58-71. 32115759 10.1002/hep.31205

[50] Tully DC, Rucker PV, Chianelli D, Williams J, Vidal A, Alper PB, et al. (2017). Discovery of Tropifexor (LJN452), a Highly Potent Non-bile Acid FXR Agonist for the Treatment of Cholestatic Liver Diseases and Nonalcoholic Steatohepatitis (NASH). J Med Chem, 60:9960-9973. 29148806 10.1021/acs.jmedchem.7b00907

[51] Fang S, Suh JM, Reilly SM, Yu E, Osborn O, Lackey D, et al. (2015). Intestinal FXR agonism promotes adipose tissue browning and reduces obesity and insulin resistance. Nat Med, 21:159-165. 25559344 10.1038/nm.3760 PMC4320010

[52] Chianelli D, Rucker PV, Roland J, Tully DC, Nelson J, Liu X, et al. (2020). Nidufexor (LMB763), a Novel FXR Modulator for the Treatment of Nonalcoholic Steatohepatitis. J Med Chem, 63:3868-3880. 31940200 10.1021/acs.jmedchem.9b01621

[53] Zheng T, Yang X, Li W, Wang Q, Chen L, Wu D, et al. (2018). Salidroside Attenuates High-Fat Diet-Induced Nonalcoholic Fatty Liver Disease via AMPK-Dependent TXNIP/NLRP3 Pathway. Oxid Med Cell Longev, 2018:8597897. 30140371 10.1155/2018/8597897 PMC6081551

[54] Li H, Xi Y, Xin X, Tian H, Hu Y (2020). Salidroside improves high-fat diet-induced non-alcoholic steatohepatitis by regulating the gut microbiota-bile acid-farnesoid X receptor axis. Biomed Pharmacother, 124:109915. 31986416 10.1016/j.biopha.2020.109915

[55] Yang S, Wei L, Xia R, Liu L, Chen Y, Zhang W, et al. (2019). Formononetin ameliorates cholestasis by regulating hepatic SIRT1 and PPARα. Biochem Biophys Res Commun, 512:770-778. 30928103 10.1016/j.bbrc.2019.03.131

[56] Yang F, Tang X, Ding L, Zhou Y, Yang Q, Gong J, et al. (2016). Curcumin protects ANIT-induced cholestasis through signaling pathway of FXR-regulated bile acid and inflammation. Sci Rep, 6:33052. 27624003 10.1038/srep33052 PMC5021964

[57] Li G, Lin W, Araya JJ, Chen T, Timmermann BN, Guo GL (2012). A tea catechin, epigallocatechin-3-gallate, is a unique modulator of the farnesoid X receptor. Toxicol Appl Pharmacol, 258:268-274. 22178739 10.1016/j.taap.2011.11.006 PMC3259191

[58] Sun L, Cai J, Gonzalez FJ (2021). The role of farnesoid X receptor in metabolic diseases, and gastrointestinal and liver cancer. Nat Rev Gastroenterol Hepatol, 18:335-347. 33568795 10.1038/s41575-020-00404-2

[59] Jia W, Wei M, Rajani C, Zheng X (2021). Targeting the alternative bile acid synthetic pathway for metabolic diseases. Protein Cell, 12:411-425. 33252713 10.1007/s13238-020-00804-9 PMC8106556

[60] Xie G, Jiang R, Wang X, Liu P, Zhao A, Wu Y, et al. (2021). Conjugated secondary 12alpha-hydroxylated bile acids promote liver fibrogenesis. EBioMedicine, 66:103290. 33752128 10.1016/j.ebiom.2021.103290 PMC8010625

[61] Huang XF, Zhao WY, Huang WD (2015). FXR and liver carcinogenesis. Acta Pharmacol Sin, 36:37-43. 25500874 10.1038/aps.2014.117 PMC4571316

[62] Chai Y, Kan L, Zhao M (2019). Enzymatic extraction optimization, anti-HBV and antioxidant activities of polysaccharides from Viscum coloratum (Kom.) Nakai. Int J Biol Macromol, 134:588-594. 31029628 10.1016/j.ijbiomac.2019.04.173

[63] Lleo A, Wang G-Q, Gershwin ME, Hirschfield GM (2020). Primary biliary cholangitis. The Lancet, 396:1915-1926. 10.1016/S0140-6736(20)31607-X 33308474

[64] Ming Y, Zhu X, Tuma-Kellner S, Ganzha A, Liebisch G, Gan-Schreier H, et al. (2019). iPla2beta Deficiency Suppresses Hepatic ER UPR, Fxr, and Phospholipids in Mice Fed with MCD Diet, Resulting in Exacerbated Hepatic Bile Acids and Biliary Cell Proliferation. Cells, 8. 31409057 10.3390/cells8080879 PMC6721660

[65] Merlen G, Ursic-Bedoya J, Jourdainne V, Kahale N, Glenisson M, Doignon I, et al. (2017). Bile acids and their receptors during liver regeneration: "Dangerous protectors". Mol Aspects Med, 56:25-33. 28302491 10.1016/j.mam.2017.03.002

[66] Manley S, Ding W (2015). Role of farnesoid X receptor and bile acids in alcoholic liver disease. Acta Pharm Sin B, 5:158-167. 26579442 10.1016/j.apsb.2014.12.011 PMC4629219

[67] Fiorucci S, Rizzo G, Donini A, Distrutti E, Santucci L (2007). Targeting farnesoid X receptor for liver and metabolic disorders. Trends Mol Med, 13:298-309. 17588816 10.1016/j.molmed.2007.06.001

[68] Xu JY, Li ZP, Zhang L, Ji G (2014). Recent insights into farnesoid X receptor in non-alcoholic fatty liver disease. World J Gastroenterol, 20:13493-13500. 25309079 10.3748/wjg.v20.i37.13493 PMC4188900

[69] Chen L, Jiao T, Liu W, Luo Y, Wang J, Guo X, et al. (2022). Hepatic cytochrome P450 8B1 and cholic acid potentiate intestinal epithelial injury in colitis by suppressing intestinal stem cell renewal. Cell Stem Cell, 29:1366-1381 e1369. 36055192 10.1016/j.stem.2022.08.008 PMC10673678

[70] Xiang J, Zhang Z, Xie H, Zhang C, Bai Y, Cao H, et al. (2021). Effect of different bile acids on the intestine through enterohepatic circulation based on FXR. Gut Microbes, 13:1949095. 34313539 10.1080/19490976.2021.1949095 PMC8346203

[71] Watanabe M HS, Wang L, Moschetta A, Mangelsdorf DJ, Heyman RA, Moore DD, Auwerx J. (2004). Bile acids lower triglyceride levels via a pathway involving FXR, SHP, and SREBP-1c. J Clin Invest, 113:1408-1418. 15146238 10.1172/JCI21025 PMC406532

[72] Clifford BL, Sedgeman LR, Williams KJ, Morand P, Cheng A, Jarrett KE, et al. (2021). FXR activation protects against NAFLD via bile-acid-dependent reductions in lipid absorption. Cell Metab, 33:1671-1684 e1674. 34270928 10.1016/j.cmet.2021.06.012 PMC8353952

[73] Fan L, Lai R, Ma N, Dong Y, Li Y, Wu Q, et al. (2021). miR-552-3p modulates transcriptional activities of FXR and LXR to ameliorate hepatic glycolipid metabolism disorder. J Hepatol, 74:8-19. 32818571 10.1016/j.jhep.2020.07.048

[74] Ploton M, Mazuy C, Gheeraert C, Dubois V, Berthier A, Dubois-Chevalier J, et al. (2018). The nuclear bile acid receptor FXR is a PKA- and FOXA2-sensitive activator of fasting hepatic gluconeogenesis. J Hepatol, 69:1099-1109. 29981427 10.1016/j.jhep.2018.06.022

[75] Albaugh VL, Banan B, Antoun J, Xiong Y, Guo Y, Ping J, et al. (2019). Role of Bile Acids and GLP-1 in Mediating the Metabolic Improvements of Bariatric Surgery. Gastroenterology, 156. 30445014 10.1053/j.gastro.2018.11.017 PMC6409186

[76] Zhang H, DiBaise JK, Zuccolo A, Kudrna D, Braidotti M, Yu Y, et al. (2009). Human gut microbiota in obesity and after gastric bypass. Proceedings of the National Academy of Sciences of the United States of America, 106:2365-2370. 19164560 10.1073/pnas.0812600106 PMC2629490

[77] Xie C JC, Shi J, Gao X, Sun D, Sun L, Wang T, Takahashi S, Anitha M, Krausz KW, Patterson AD, Gonzalez FJ. (2017). An Intestinal Farnesoid X Receptor-Ceramide Signaling Axis Modulates Hepatic Gluconeogenesis in Mice. Diabetes. , 66:613-626. 28223344 10.2337/db16-0663 PMC5319721

[78] Faith JJ, Guruge JL, Charbonneau M, Subramanian S, Seedorf H, Goodman AL, et al. (2013). The long-term stability of the human gut microbiota. Science, 341:1237439. 23828941 10.1126/science.1237439 PMC3791589

[79] Qin J, Li R, Raes J, Arumugam M, Burgdorf KS, Manichanh C, et al. (2010). A human gut microbial gene catalogue established by metagenomic sequencing. Nature, 464:59-65. 20203603 10.1038/nature08821 PMC3779803

[80] HMP Consortium. (2012). Structure, function and diversity of the healthy human microbiome. Nature., 486:207-214. 22699609 10.1038/nature11234 PMC3564958

[81] Bao W, He Y, Yu J, Liu M, Yang X, Ta N, et al. (2022). Regulatory Effect of Lactiplantibacillus plantarum 2-33 on Intestinal Microbiota of Mice With Antibiotic-Associated Diarrhea. Front Nutr, 9:921875. 35757257 10.3389/fnut.2022.921875 PMC9218693

[82] Ma X, Bi Q, Kong Y, Xu H, Liang M, Mai K, et al. (2022). Dietary lipid levels affected antioxidative status, inflammation response, apoptosis and microbial community in the intestine of juvenile turbot (Scophthalmus maximus L.). Comp Biochem Physiol A Mol Integr Physiol, 264:111118. 34793954 10.1016/j.cbpa.2021.111118

[83] Xiao X, Cheng Y, Fu J, Lu Z, Wang F, Jin M, et al. (2021). Gut Immunity and Microbiota Dysbiosis Are Associated with Altered Bile Acid Metabolism in LPS-Challenged Piglets. Oxid Med Cell Longev, 2021:6634821. 33833852 10.1155/2021/6634821 PMC8018853

[84] Jian YP, Yang G, Zhang LH, Liang JY, Zhou HL, Wang YS, et al. (2022). Lactobacillus plantarum alleviates irradiation-induced intestinal injury by activation of FXR-FGF15 signaling in intestinal epithelia. J Cell Physiol, 237:1845-1856. 34881818 10.1002/jcp.30651

[85] Inagaki T MA, Lee YK, Peng L, Zhao G, Downes M, Yu RT, Shelton JM, Richardson JA, Repa JJ, Mangelsdorf DJ, Kliewer SA. (2006). Regulation of antibacterial defense in the small intestine by the nuclear bile acid receptor. Proc Natl Acad Sci U S A, 103:3920-3925. 16473946 10.1073/pnas.0509592103 PMC1450165

[86] Wiest R, Lawson M, Geuking M (2014). Pathological bacterial translocation in liver cirrhosis. J Hepatol, 60:197-209. 23993913 10.1016/j.jhep.2013.07.044

[87] Sorribas M, Jakob MO, Yilmaz B, Li H, Stutz D, Noser Y, et al. (2019). FXR modulates the gut-vascular barrier by regulating the entry sites for bacterial translocation in experimental cirrhosis. J Hepatol, 71:1126-1140. 31295531 10.1016/j.jhep.2019.06.017

[88] Parseus A, Sommer N, Sommer F, Caesar R, Molinaro A, Stahlman M, et al. (2017). Microbiota-induced obesity requires farnesoid X receptor. Gut, 66:429-437. 26740296 10.1136/gutjnl-2015-310283 PMC5534765

[89] Shu X, Li M, Cao Y, Li C, Zhou W, Ji G, et al. (2021). Berberine Alleviates Non-alcoholic Steatohepatitis Through Modulating Gut Microbiota Mediated Intestinal FXR Activation. Front Pharmacol, 12:750826. 34603061 10.3389/fphar.2021.750826 PMC8484326

[90] Guzior DV, Quinn RA (2021). Review: microbial transformations of human bile acids. Microbiome, 9:140. 34127070 10.1186/s40168-021-01101-1 PMC8204491

[91] Huang F, Zheng X, Ma X, Jiang R, Zhou W, Zhou S, et al. (2019). Theabrownin from Pu-erh tea attenuates hypercholesterolemia via modulation of gut microbiota and bile acid metabolism. Nat Commun, 10:4971. 31672964 10.1038/s41467-019-12896-x PMC6823360

[92] Groen AK, Bloks VW, Verkade H, Kuipers F (2014). Cross-talk between liver and intestine in control of cholesterol and energy homeostasis. Mol Aspects Med, 37:77-88. 24560594 10.1016/j.mam.2014.02.001

[93] He B, Jiang J, Shi Z, Wu L, Yan J, Chen Z, et al. (2021). Pure total flavonoids from citrus attenuate non-alcoholic steatohepatitis via regulating the gut microbiota and bile acid metabolism in mice. Biomed Pharmacother, 135:111183. 33401222 10.1016/j.biopha.2020.111183

[94] Xue R, Su L, Lai S, Wang Y, Zhao D, Fan J, et al. (2021). Bile Acid Receptors and the Gut-Liver Axis in Nonalcoholic Fatty Liver Disease. Cells, 10. 34831031 10.3390/cells10112806 PMC8616422

[95] Gonzalez FJ (2012). Nuclear receptor control of enterohepatic circulation. Compr Physiol, 2:2811-2828. 23720266 10.1002/cphy.c120007 PMC6608752

[96] Beaudoin JJ, Brouwer KLR, Malinen MM (2020). Novel insights into the organic solute transporter alpha/beta, OSTalpha/beta: From the bench to the bedside. Pharmacol Ther, 211:107542. 32247663 10.1016/j.pharmthera.2020.107542 PMC7480074

[97] Hwang ST, Urizar NL, Moore DD, Henning SJ (2002). Bile acids regulate the ontogenic expression of ileal bile acid binding protein in the rat via the farnesoid X receptor. Gastroenterology, 122:1483-1492. 11984532 10.1053/gast.2002.32982

[98] Keely SJ, Walters JR (2016). The Farnesoid X Receptor: Good for BAD. Cell Mol Gastroenterol Hepatol, 2:725-732. 28174746 10.1016/j.jcmgh.2016.08.004 PMC5247348

[99] Degirolamo C, Rainaldi S, Bovenga F, Murzilli S, Moschetta A (2014). Microbiota modification with probiotics induces hepatic bile acid synthesis via downregulation of the Fxr-Fgf15 axis in mice. Cell Rep, 7:12-18. 24656817 10.1016/j.celrep.2014.02.032

[100] Bennett BJ, de Aguiar Vallim TQ, Wang Z, Shih DM, Meng Y, Gregory J, et al. (2013). Trimethylamine-N-oxide, a metabolite associated with atherosclerosis, exhibits complex genetic and dietary regulation. Cell Metab, 17:49-60. 23312283 10.1016/j.cmet.2012.12.011 PMC3771112

[101] Ito K, Okumura A, Takeuchi JS, Watashi K, Inoue R, Yamauchi T, et al. (2021). Dual Agonist of Farnesoid X Receptor and Takeda G Protein-Coupled Receptor 5 Inhibits Hepatitis B Virus Infection In Vitro and In Vivo. Hepatology, 74:83-98. 33434356 10.1002/hep.31712

[102] Chen X, Meng Q, Wang C, Liu Q, Sun H, Huo X, et al. (2015). Protective effects of calycosin against CCl4-induced liver injury with activation of FXR and STAT3 in mice. Pharm Res, 32:538-548. 25143196 10.1007/s11095-014-1483-3

[103] Hartmann P, Hochrath K, Horvath A, Chen P, Seebauer CT, Llorente C, et al. (2018). Modulation of the intestinal bile acid/farnesoid X receptor/fibroblast growth factor 15 axis improves alcoholic liver disease in mice. Hepatology, 67:2150-2166. 29159825 10.1002/hep.29676 PMC5962369

[104] Massafra V, Pellicciari R, Gioiello A, van Mil SWC (2018). Progress and challenges of selective Farnesoid X Receptor modulation. Pharmacol Ther, 191:162-177. 29933033 10.1016/j.pharmthera.2018.06.009

[105] Wu W, Zhu B, Peng X, Zhou M, Jia D, Gu J (2014). Activation of farnesoid X receptor attenuates hepatic injury in a murine model of alcoholic liver disease. Biochem Biophys Res Commun, 443:68-73. 24269813 10.1016/j.bbrc.2013.11.057

[106] Wang S, Sheng F, Zou L, Xiao J, Li P (2021). Hyperoside attenuates non-alcoholic fatty liver disease in rats via cholesterol metabolism and bile acid metabolism. J Adv Res, 34:109-122. 35024184 10.1016/j.jare.2021.06.001 PMC8655136

[107] Tanaka N, Aoyama T, Kimura S, Gonzalez FJ (2017). Targeting nuclear receptors for the treatment of fatty liver disease. Pharmacol Ther, 179:142-157. 28546081 10.1016/j.pharmthera.2017.05.011 PMC6659998

[108] Levy G, Habib N, Guzzardi MA, Kitsberg D, Bomze D, Ezra E, et al. (2016). Nuclear receptors control pro-viral and antiviral metabolic responses to hepatitis C virus infection. Nat Chem Biol, 12:1037-1045. 27723751 10.1038/nchembio.2193 PMC7046487

[109] Levy G, Habib N, Guzzardi MA, Kitsberg D, Bomze D, Ezra E, et al. (2016). Nuclear receptors control pro-viral and antiviral metabolic responses to hepatitis C virus infection. Nature Chemical Biology, 12:1037-1045. 27723751 10.1038/nchembio.2193 PMC7046487

[110] Lu W, Cheng F, Jiang J, Zhang C, Deng X, Xu Z, et al. (2015). FXR antagonism of NSAIDs contributes to drug-induced liver injury identified by systems pharmacology approach. Sci Rep, 5:8114. 25631039 10.1038/srep08114 PMC4310094

[111] Han X, Cui Z-Y, Song J, Piao H-Q, Lian L-H, Hou L-S, et al. (2019). Acanthoic acid modulates lipogenesis in nonalcoholic fatty liver disease via FXR/LXRs-dependent manner. Chemico-Biological Interactions, 311. 10.1016/j.cbi.2019.108794 31421115

[112] Cui ZY, Han X, Jiang YC, Dou JY, Yao KC, Hu ZH, et al. (2021). Allium victorialis L. Extracts Promote Activity of FXR to Ameliorate Alcoholic Liver Disease: Targeting Liver Lipid Deposition and Inflammation. Front Pharmacol, 12:738689. 34690775 10.3389/fphar.2021.738689 PMC8531498

[113] Pellicciari R, Fiorucci S, Camaioni E, Clerici C, Costantino G, Maloney PR, et al. (2002). 6alpha-ethyl-chenodeoxycholic acid (6-ECDCA), a potent and selective FXR agonist endowed with anticholestatic activity. Journal of Medicinal Chemistry, 45:3569-3572. 12166927 10.1021/jm025529g

[114] Fiorucci S, Clerici C, Antonelli E, Orlandi S, Goodwin B, Sadeghpour BM, et al. (2005). Protective effects of 6-ethyl chenodeoxycholic acid, a farnesoid X receptor ligand, in estrogen-induced cholestasis. The Journal of Pharmacology and Experimental Therapeutics, 313:604-612. 15644430 10.1124/jpet.104.079665

[115] Rizzo G, Disante M, Mencarelli A, Renga B, Gioiello A, Pellicciari R, et al. (2006). The farnesoid X receptor promotes adipocyte differentiation and regulates adipose cell function in vivo. Molecular Pharmacology, 70:1164-1173. 16778009 10.1124/mol.106.023820

[116] Wu J, Masuy I, Biesiekierski JR, Fitzke HE, Parikh C, Schofield L, et al. (2022). Gut-brain axis dysfunction underlies FODMAP-induced symptom generation in irritable bowel syndrome. Aliment Pharmacol Ther, 55:670-682. 35166384 10.1111/apt.16812

[117] Li M, Tan HE, Lu Z, Tsang KS, Chung AJ, Zuker CS (2022). Gut-Brain Circuits for Fat Preference. Nature. 10.1038/s41586-022-05266-z PMC9605869 36070796

[118] Rivera LR, Leung C, Pustovit RV, Hunne BL, Andrikopoulos S, Herath C, et al. (2014). Damage to enteric neurons occurs in mice that develop fatty liver disease but not diabetes in response to a high-fat diet. Neurogastroenterology & Motility, 26:1188-1199. 24952996 10.1111/nmo.12385

[119] Xu M-Y, Guo C-C, Li M-Y, Lou Y-H, Chen Z-R, Liu B-W, et al. (2022). Brain-gut-liver axis: Chronic psychological stress promotes liver injury and fibrosis via gut in rats. Frontiers in Cellular and Infection Microbiology, 12:1040749. 36579341 10.3389/fcimb.2022.1040749 PMC9791198

[120] Kim CS, Cha L, Sim M, Jung S, Chun WY, Baik HW, et al. (2021). Probiotic Supplementation Improves Cognitive Function and Mood with Changes in Gut Microbiota in Community-Dwelling Older Adults: A Randomized, Double-Blind, Placebo-Controlled, Multicenter Trial. J Gerontol A Biol Sci Med Sci, 76:32-40. 32300799 10.1093/gerona/glaa090 PMC7861012

[121] Margolis KG, Cryan JF, Mayer EA (2021). The Microbiota-Gut-Brain Axis: From Motility to Mood. Gastroenterology, 160:1486-1501. 33493503 10.1053/j.gastro.2020.10.066 PMC8634751

[122] Liu S, Marcelin G, Blouet C, Jeong JH, Jo YH, Schwartz GJ, et al. (2017). A gut-brain axis regulating glucose metabolism mediated by bile acids and competitive fibroblast growth factor actions at the hypothalamus. Mol Metab, 8:37-50. 29290621 10.1016/j.molmet.2017.12.003 PMC5985052

[123] Jena PK, Sheng L, Nguyen M, Di Lucente J, Hu Y, Li Y, et al. (2020). Dysregulated bile acid receptor-mediated signaling and IL-17A induction are implicated in diet-associated hepatic health and cognitive function. Biomark Res. 8:59. 33292701 10.1186/s40364-020-00239-8 PMC7648397

[124] Liu F, Yao Y, Wang Q, Zhang F, Wang M, Zhu C, et al. (2022). Nigakinone alleviates DSS-induced experimental colitis via regulating bile acid profile and FXR/NLRP3 signaling pathways. Phytother Res, 37:15-34. 36054406 10.1002/ptr.7588

[125] Bao H, Li H, Jia Y, Xiao Y, Luo S, Zhang D, et al. (2021). Ganoderic acid A exerted antidepressant-like action through FXR modulated NLRP3 inflammasome and synaptic activity. Biochem Pharmacol, 188:114561. 33857491 10.1016/j.bcp.2021.114561

[126] Seki E, Brenner DA (2015). Recent advancement of molecular mechanisms of liver fibrosis. J Hepatobiliary Pancreat Sci, 22:512-518. 25869468 10.1002/jhbp.245 PMC4668270

[127] Bottcher K, Pinzani M (2017). Pathophysiology of liver fibrosis and the methodological barriers to the development of anti-fibrogenic agents. Adv Drug Deliv Rev, 121:3-8. 28600202 10.1016/j.addr.2017.05.016

[128] van Grunsven LA (2017). 3D in vitro models of liver fibrosis. Adv Drug Deliv Rev, 121:133-146. 28697953 10.1016/j.addr.2017.07.004

[129] Teare JP, Sherman D, Greenfield SM, Simpson J, Bray G, Catterall AP, et al. (1993). Comparison of serum procollagen III peptide concentrations and PGA index for assessment of hepatic fibrosis. Lancet (London, England), 342:895-898. 8105167 10.1016/0140-6736(93)91946-j

[130] Jiang JJ, Salvucci M, Thepot V, Pol S, Ekindjian OG, Nalpas B (1994). PGA score in diagnosis of alcoholic fibrosis. Lancet (London, England), 343:803. 10.1016/s0140-6736(94)91887-2 7907768

[131] Suzuki A, Angulo P, Lymp J, Li D, Satomura S, Lindor K (2005). Hyaluronic acid, an accurate serum marker for severe hepatic fibrosis in patients with non-alcoholic fatty liver disease. Liver International : Official Journal of the International Association For the Study of the Liver, 25:779-786. 15998429 10.1111/j.1478-3231.2005.01064.x

[132] Harrison SA, Ratziu V, Boursier J, Francque S, Bedossa P, Majd Z, et al. (2020). A blood-based biomarker panel (NIS4) for non-invasive diagnosis of non-alcoholic steatohepatitis and liver fibrosis: a prospective derivation and global validation study. The Lancet. Gastroenterology & Hepatology, 5:970-985. 32763196 10.1016/S2468-1253(20)30252-1

[133] Lu L, G ZM, Mao YM, Li JQ, Qiu DK, Fang JY, et al. (2003). Relationship between clinical and pathologic findings in patients with chronic liver diseases. World J Gastroenterol, 9:2796-2800. 14669336 10.3748/wjg.v9.i12.2796 PMC4612055

[134] Aizarani N, Saviano A, Sagar, Mailly L, Durand S, Herman JS, et al. (2019). A human liver cell atlas reveals heterogeneity and epithelial progenitors. Nature, 572:199-204. 31292543 10.1038/s41586-019-1373-2 PMC6687507

[135] Henderson NC, Rieder F, Wynn TA (2020). Fibrosis: from mechanisms to medicines. Nature, 587:555-566. 33239795 10.1038/s41586-020-2938-9 PMC8034822

[136] Campana L, Iredale JP (2017). Regression of Liver Fibrosis. Semin Liver Dis, 37:1-10. 28201843 10.1055/s-0036-1597816

[137] Campana L, Esser H, Huch M, Forbes S (2021). Liver regeneration and inflammation: from fundamental science to clinical applications. Nat Rev Mol Cell Biol, 22:608-624. 34079104 10.1038/s41580-021-00373-7

[138] Roeb E (2018). Matrix metalloproteinases and liver fibrosis (translational aspects). Matrix Biol, 68-69:463-473. 29289644 10.1016/j.matbio.2017.12.012

[139] Wang J, Kubes P (2016). A Reservoir of Mature Cavity Macrophages that Can Rapidly Invade Visceral Organs to Affect Tissue Repair. Cell, 165:668-678. 27062926 10.1016/j.cell.2016.03.009

[140] Xu M, Xu HH, Lin Y, Sun X, Wang LJ, Fang ZP, et al. (2019). LECT2, a Ligand for Tie1, Plays a Crucial Role in Liver Fibrogenesis. Cell, 178:1478-1492 e1420. 31474362 10.1016/j.cell.2019.07.021

[141] Friedman SL, Pinzani M (2022). Hepatic fibrosis 2022: Unmet needs and a blueprint for the future. Hepatology (Baltimore, Md.), 75:473-488. 10.1002/hep.32285 PMC12179971 34923653

[142] Ajmera V, Loomba R (2021). Imaging biomarkers of NAFLD, NASH, and fibrosis. Molecular Metabolism, 50:101167. 33460786 10.1016/j.molmet.2021.101167 PMC8324681

[143] Angelini G, Panunzi S, Castagneto-Gissey L, Pellicanò F, De Gaetano A, Pompili M, et al. (2023). Accurate liquid biopsy for the diagnosis of non-alcoholic steatohepatitis and liver fibrosis. Gut, 72:392-403. 35820779 10.1136/gutjnl-2022-327498 PMC9872242

[144] Sanyal AJ, Foucquier J, Younossi ZM, Harrison SA, Newsome PN, Chan W-K, et al. (2023). Enhanced diagnosis of advanced fibrosis and cirrhosis in individuals with NAFLD using FibroScan-based Agile scores. Journal of Hepatology, 78:247-259. 36375686 10.1016/j.jhep.2022.10.034 PMC10170177

[145] Massey V, Parrish A, Argemi J, Moreno M, Mello A, Garcia-Rocha M, et al. (2021). Integrated Multiomics Reveals Glucose Use Reprogramming and Identifies a Novel Hexokinase in Alcoholic Hepatitis. Gastroenterology, 160:1725-1740 e1722. 33309778 10.1053/j.gastro.2020.12.008 PMC8613537

[146] Kisseleva T, Brenner D (2021). Molecular and cellular mechanisms of liver fibrosis and its regression. Nat Rev Gastroenterol Hepatol, 18:151-166. 33128017 10.1038/s41575-020-00372-7

[147] Xu W, Cui C, Cui C, Chen Z, Zhang H, Cui Q, et al. (2022). Hepatocellular Cystathionine gamma lyase/Hydrogen sulfide Attenuates Non-Alcoholic Fatty Liver Disease by Activating Farnesoid X Receptor. Hepatology. 10.1002/hep.32577 PMC9795901 35586979

[148] Xu W, Cui C, Cui C, Chen Z, Zhang H, Cui Q, et al. (2022). Hepatocellular cystathionine gamma lyase/hydrogen sulfide attenuates nonalcoholic fatty liver disease by activating farnesoid X receptor. Hepatology, 76:1794-1810. 35586979 10.1002/hep.32577 PMC9795901

[149] Liu S, Qin D, Yan Y, Wu J, Meng L, Huang W, et al. (2021). Metabolic nuclear receptors coordinate energy metabolism to regulate Sox9(+) hepatocyte fate. iScience, 24:103003. 34505013 10.1016/j.isci.2021.103003 PMC8417399

[150] Han X, Wang Y, Pu W, Huang X, Qiu L, Li Y, et al. (2019). Lineage Tracing Reveals the Bipotency of SOX9(+) Hepatocytes during Liver Regeneration. Stem Cell Reports, 12:624-638. 30773487 10.1016/j.stemcr.2019.01.010 PMC6409431

[151] Borude P, Edwards G, Walesky C, Li F, Ma X, Kong B, et al. (2012). Hepatocyte-specific deletion of farnesoid X receptor delays but does not inhibit liver regeneration after partial hepatectomy in mice. Hepatology, 56:2344-2352. 22730081 10.1002/hep.25918 PMC3469721

[152] Ratziu V, Rinella ME, Neuschwander-Tetri BA, Lawitz E, Denham D, Kayali Z, et al. (2022). EDP-305 in patients with NASH: A phase II double-blind placebo-controlled dose-ranging study. J Hepatol, 76:506-517. 34740705 10.1016/j.jhep.2021.10.018

[153] Erken R, Andre P, Roy E, Kootstra N, Barzic N, Girma H, et al. (2021). Farnesoid X receptor agonist for the treatment of chronic hepatitis B: A safety study. J Viral Hepat, 28:1690-1698. 34467593 10.1111/jvh.13608 PMC9293351

[154] Bieghs V, Trautwein C (2013). The innate immune response during liver inflammation and metabolic disease. Trends Immunol, 34:446-452. 23668977 10.1016/j.it.2013.04.005

[155] Shuai Z, Leung MW, He X, Zhang W, Yang G, Leung PS, et al. (2016). Adaptive immunity in the liver. Cellular & Molecular Immunology, 13:354-368. 26996069 10.1038/cmi.2016.4 PMC4856810

[156] Zhang Q, Raoof M, Chen Y, Sumi Y, Sursal T, Junger W, et al. (2010). Circulating mitochondrial DAMPs cause inflammatory responses to injury. Nature, 464:104-107. 20203610 10.1038/nature08780 PMC2843437

[157] Dixon LJ, Barnes M, Tang H, Pritchard MT, Nagy LE (2013). Kupffer cells in the liver. Comprehensive Physiology, 3:785-797. 23720329 10.1002/cphy.c120026 PMC4748178

[158] Fiorucci S, Baldoni M, Ricci P, Zampella A, Distrutti E, Biagioli M (2020). Bile acid-activated receptors and the regulation of macrophages function in metabolic disorders. Curr Opin Pharmacol, 53:45-54. 32480317 10.1016/j.coph.2020.04.008

[159] Eming SA, Wynn TA, Martin P (2017). Inflammation and metabolism in tissue repair and regeneration. Science, 356:1026-1030. 28596335 10.1126/science.aam7928

[160] Racanelli V, Rehermann B (2006). The liver as an immunological organ. Hepatology (Baltimore, Md.), 43:S54-S62. 10.1002/hep.21060 16447271

[161] Vavassori P, Mencarelli A, Renga B, Distrutti E, Fiorucci S (2009). The Bile Acid Receptor FXR Is a Modulator of Intestinal Innate Immunity. The Journal of Immunology, 183:6251-6261. 19864602 10.4049/jimmunol.0803978

[162] Yuk J-M, Shin D-M, Lee H-M, Kim J-J, Kim S-W, Jin HS, et al. (2011). The orphan nuclear receptor SHP acts as a negative regulator in inflammatory signaling triggered by Toll-like receptors. Nature Immunology, 12:742-751. 21725320 10.1038/ni.2064

[163] Jin D, Lu T, Ni M, Wang H, Zhang J, Zhong C, et al. (2020). Farnesoid X Receptor Activation Protects Liver From Ischemia/Reperfusion Injury by Up-Regulating Small Heterodimer Partner in Kupffer Cells. Hepatology Communications, 4:540-554. 32258949 10.1002/hep4.1478 PMC7109340

[164] Hucke S, Herold M, Liebmann M, Freise N, Lindner M, Fleck A-K, et al. (2016). The farnesoid-X-receptor in myeloid cells controls CNS autoimmunity in an IL-10-dependent fashion. Acta Neuropathologica, 132:413-431. 27383204 10.1007/s00401-016-1593-6

[165] Hao H, Cao L, Jiang C, Che Y, Zhang S, Takahashi S, et al. (2017). Farnesoid X Receptor Regulation of the NLRP3 Inflammasome Underlies Cholestasis-Associated Sepsis. Cell Metab, 25:856-867 e855. 28380377 10.1016/j.cmet.2017.03.007 PMC6624427

[166] Huang S, Wu Y, Zhao Z, Wu B, Sun K, Wang H, et al. (2021). A new mechanism of obeticholic acid on NASH treatment by inhibiting NLRP3 inflammasome activation in macrophage. Metabolism, 120:154797. 33984334 10.1016/j.metabol.2021.154797

[167] Mi L-Z, Devarakonda S, Harp JM, Han Q, Pellicciari R, Willson TM, et al. (2003). Structural basis for bile acid binding and activation of the nuclear receptor FXR. Molecular Cell, 11:1093-1100. 12718893 10.1016/s1097-2765(03)00112-6

[168] Capote J, Kramerova I, Martinez L, Vetrone S, Barton ER, Sweeney HL, et al. (2016). Osteopontin ablation ameliorates muscular dystrophy by shifting macrophages to a pro-regenerative phenotype. J Cell Biol, 213:275-288. 27091452 10.1083/jcb.201510086 PMC5084275

[169] Mencarelli A, Renga B, Migliorati M, Cipriani S, Distrutti E, Santucci L, et al. (2009). The bile acid sensor farnesoid X receptor is a modulator of liver immunity in a rodent model of acute hepatitis. J Immunol, 183:6657-6666. 19880446 10.4049/jimmunol.0901347

[170] Ma C, Han M, Heinrich B, Fu Q, Zhang Q, Sandhu M, et al. (2018). Gut microbiome-mediated bile acid metabolism regulates liver cancer via NKT cells. Science, 360. 10.1126/science.aan5931 PMC6407885 29798856

[171] Gou H, Liu S, Liu L, Luo M, Qin S, He K, et al. (2022). Obeticholic acid and 5β-cholanic acid 3 exhibit anti-tumor effects on liver cancer through CXCL16/CXCR6 pathway. Frontiers in Immunology, 13. 1095915. 36605219 10.3389/fimmu.2022.1095915 PMC9807878

[172] Meadows V, Kennedy L, Ekser B, Kyritsi K, Kundu D, Zhou T, et al. (2021). Mast Cells Regulate Ductular Reaction and Intestinal Inflammation in Cholestasis Through Farnesoid X Receptor Signaling. Hepatology, 74:2684-2698. 34164827 10.1002/hep.32028 PMC9337218

[173] Fiorucci S, Zampella A, Ricci P, Distrutti E, Biagioli M (2022). Immunomodulatory functions of FXR. Molecular and Cellular Endocrinology, 551. 10.1016/j.mce.2022.111650 35472625

[174] Puche JE, Saiman Y, Friedman SL (2013). Hepatic stellate cells and liver fibrosis. Compr Physiol, 3:1473-1492. 24265236 10.1002/cphy.c120035

[175] Saeed A, Hoekstra M, Hoeke MO, Heegsma J, Faber KN (2017). The interrelationship between bile acid and vitamin A homeostasis. Biochim Biophys Acta Mol Cell Biol Lipids, 1862:496-512. 28111285 10.1016/j.bbalip.2017.01.007

[176] Saeed A, Yang J, Heegsma J, Groen AK, van Mil SWC, Paulusma CC, et al. (2019). Farnesoid X receptor and bile acids regulate vitamin A storage. Sci Rep, 9:19493. 31862954 10.1038/s41598-019-55988-w PMC6925179

[177] Fiorucci S, Antonelli E, Rizzo G, Renga B, Mencarelli A, Riccardi L, et al. (2004). The nuclear receptor SHP mediates inhibition of hepatic stellate cells by FXR and protects against liver fibrosis. Gastroenterology, 127:1497-1512. 15521018 10.1053/j.gastro.2004.08.001

[178] Vogel S, Piantedosi R, Frank J, Lalazar A, Rockey DC, Friedman SL, et al. (2000). An immortalized rat liver stellate cell line (HSC-T6): a new cell model for the study of retinoid metabolism in vitro. Journal of Lipid Research, 41:882-893. 10828080

[179] Forman BM, Goode E, Chen J, Oro AE, Bradley DJ, Perlmann T, et al. (1995). Identification of a nuclear receptor that is activated by farnesol metabolites. Cell, 81:687-693. 7774010 10.1016/0092-8674(95)90530-8

[180] Parks DJ, Blanchard SG, Bledsoe RK, Chandra G, Consler TG, Kliewer SA, et al. (1999). Bile acids: natural ligands for an orphan nuclear receptor. Science (New York, N.Y.), 284:1365-1368. 10334993 10.1126/science.284.5418.1365

[181] Kim KH, Choi S, Zhou Y, Kim EY, Lee JM, Saha PK, et al. (2017). Hepatic FXR/SHP axis modulates systemic glucose and fatty acid homeostasis in aged mice. Hepatology (Baltimore, Md.), 66:498-509. 10.1002/hep.29199 PMC8156739 28378930

[182] Gong J, Yang F, Yang Q, Tang X, Shu F, Xu L, et al. (2020). Sweroside ameliorated carbon tetrachloride (CCl4)-induced liver fibrosis through FXR-miR-29a signaling pathway. J Nat Med, 74:17-25. 31280460 10.1007/s11418-019-01334-3

[183] Tsuchida T, Friedman SL (2017). Mechanisms of hepatic stellate cell activation. Nat Rev Gastroenterol Hepatol, 14:397-411. 28487545 10.1038/nrgastro.2017.38

[184] Carino A, Biagioli M, Marchiano S, Scarpelli P, Zampella A, Limongelli V, et al. (2018). Disruption of TFGbeta-SMAD3 pathway by the nuclear receptor SHP mediates the antifibrotic activities of BAR704, a novel highly selective FXR ligand. Pharmacol Res, 131:17-31. 29530598 10.1016/j.phrs.2018.02.033

[185] Xu W, Lu C, Zhang F, Shao J, Zheng S (2016). Dihydroartemisinin restricts hepatic stellate cell contraction via an FXR-S1PR2-dependent mechanism. IUBMB Life, 68:376-387. 27027402 10.1002/iub.1492

[186] Chopyk DM, Grakoui A (2020). Contribution of the Intestinal Microbiome and Gut Barrier to Hepatic Disorders. Gastroenterology, 159:849-863. 32569766 10.1053/j.gastro.2020.04.077 PMC7502510

[187] Bailey MA, Holscher HD (2018). Microbiome-Mediated Effects of the Mediterranean Diet on Inflammation. Adv Nutr, 9:193-206. 29767701 10.1093/advances/nmy013 PMC5952955

[188] Schwimmer JB, Johnson JS, Angeles JE, Behling C, Belt PH, Borecki I, et al. (2019). Microbiome Signatures Associated With Steatohepatitis and Moderate to Severe Fibrosis in Children With Nonalcoholic Fatty Liver Disease. Gastroenterology, 157:1109-1122. 31255652 10.1053/j.gastro.2019.06.028 PMC6756995

[189] Iljazovic A, Roy U, Galvez EJC, Lesker TR, Zhao B, Gronow A, et al. (2021). Perturbation of the gut microbiome by Prevotella spp. enhances host susceptibility to mucosal inflammation. Mucosal Immunol, 14:113-124. 32433514 10.1038/s41385-020-0296-4 PMC7790746

[190] Ley RE (2016). Gut microbiota in 2015: Prevotella in the gut: choose carefully. Nat Rev Gastroenterol Hepatol, 13:69-70. 26828918 10.1038/nrgastro.2016.4

[191] Guan M, Pan D, Zhang M, Leng X, Yao B, Elvy Suhana MR (2021). The Aqueous Extract of Eucommia Leaves Promotes Proliferation, Differentiation, and Mineralization of Osteoblast-Like MC3T3-E1 Cells. Evidence-Based Complementary and Alternative Medicine, 2021:1-12. 10.1155/2021/3641317 PMC8238580 34249129

[192] Liu S, Kang W, Mao X, Ge L, Du H, Li J, et al. (2022). Melatonin mitigates aflatoxin B1-induced liver injury via modulation of gut microbiota/intestinal FXR/liver TLR4 signaling axis in mice. J Pineal Res, 73:e12812. 35652241 10.1111/jpi.12812

[193] Jiang B, Yuan G, Wu J, Wu Q, Li L, Jiang P (2022). Prevotella copri ameliorates cholestasis and liver fibrosis in primary sclerosing cholangitis by enhancing the FXR signalling pathway. Biochim Biophys Acta Mol Basis Dis, 1868:166320. 34896545 10.1016/j.bbadis.2021.166320

[194] Pean N, Le Lay A, Brial F, Wasserscheid J, Rouch C, Vincent M, et al. (2020). Dominant gut Prevotella copri in gastrectomised non-obese diabetic Goto-Kakizaki rats improves glucose homeostasis through enhanced FXR signalling. Diabetologia, 63:1223-1235. 32173762 10.1007/s00125-020-05122-7 PMC7228998

[195] Cho YE, Yu LR, Abdelmegeed MA, Yoo SH, Song BJ (2018). Apoptosis of enterocytes and nitration of junctional complex proteins promote alcohol-induced gut leakiness and liver injury. J Hepatol, 69:142-153. 29458168 10.1016/j.jhep.2018.02.005 PMC6008177

[196] Mir H, Meena AS, Chaudhry KK, Shukla PK, Gangwar R, Manda B, et al. (2016). Occludin deficiency promotes ethanol-induced disruption of colonic epithelial junctions, gut barrier dysfunction and liver damage in mice. Biochim Biophys Acta, 1860:765-774. 26721332 10.1016/j.bbagen.2015.12.013 PMC4776745

[197] Song M, Ye J, Zhang F, Su H, Yang X, He H, et al. (2019). Chenodeoxycholic Acid (CDCA) Protects against the Lipopolysaccharide-Induced Impairment of the Intestinal Epithelial Barrier Function via the FXR-MLCK Pathway. J Agric Food Chem, 67:8868-8874. 31319027 10.1021/acs.jafc.9b03173

[198] Liu Y CK, Li F, Gu Z, Liu Q, He L, Shao T, Song Q, Zhu F, Zhang L, Jiang M, Zhou Y, Barve S, Zhang X, McClain CJ, Feng W. (2020). Probiotic Lactobacillus rhamnosus GG Prevents Liver Fibrosis Through Inhibiting Hepatic Bile Acid Synthesis and Enhancing Bile Acid Excretion in Mice. Hepatology, 71:2050-2066. 31571251 10.1002/hep.30975 PMC7317518

[199] Gonzalez FJ, Jiang C, Xie C, Patterson AD (2017). Intestinal Farnesoid X Receptor Signaling Modulates Metabolic Disease. Dig Dis, 35:178-184. 28249275 10.1159/000450908 PMC6595218

[200] Mak PA, Kast-Woelbern HR, Anisfeld AM, Edwards PA (2002). Identification of PLTP as an LXR target gene and apoE as an FXR target gene reveals overlapping targets for the two nuclear receptors. Journal of Lipid Research, 43:2037-2041. 12454263 10.1194/jlr.c200014-jlr200

[201] Byun S, Jung H, Chen J, Kim Y-C, Kim D-H, Kong B, et al. (2019). Phosphorylation of hepatic farnesoid X receptor by FGF19 signaling-activated Src maintains cholesterol levels and protects from atherosclerosis. Journal of Biological Chemistry, 294:8732-8744. 30996006 10.1074/jbc.RA119.008360 PMC6552419

[202] Kim H-J, Kim J-Y, Kim J-Y, Park S-K, Seo J-H, Kim JB, et al. (2004). Differential Regulation of Human and Mouse Orphan Nuclear Receptor Small Heterodimer Partner Promoter by Sterol Regulatory Element Binding Protein-1. Journal of Biological Chemistry, 279:28122-28131. 15123650 10.1074/jbc.M313302200

[203] Liu S, Kang W, Mao X, Ge L, Du H, Li J, et al. (2022). Melatonin mitigates aflatoxin B1-induced liver injury via modulation of gut microbiota/intestinal FXR/liver TLR4 signaling axis in mice. J Pineal Res: e12812. 35652241 10.1111/jpi.12812

[204] Petrov PD, Soluyanova P, Sánchez-Campos S, Castell JV, Jover R (2021). Molecular mechanisms of hepatotoxic cholestasis by clavulanic acid: Role of NRF2 and FXR pathways. Food and Chemical Toxicology, 158 :112664. 34767876 10.1016/j.fct.2021.112664

[205] Liu C, Pan Z, Wu Z, Tang K, Zhong Y, Chen Y, et al. (2022). Hepatic SIRT6 Modulates Transcriptional Activities of FXR to Alleviate Acetaminophen-induced Hepatotoxicity. Cell Mol Gastroenterol Hepatol, 14:271-293. 35526796 10.1016/j.jcmgh.2022.04.011 PMC9218579

[206] Ma Y, Liu X, Liu D, Yin Z, Yang X, Zeng M (2022). Oyster (Crassostrea gigas) Polysaccharide Ameliorates High-Fat-Diet-Induced Oxidative Stress and Inflammation in the Liver via the Bile Acid-FXR-AMPKα Pathway. Journal of Agricultural and Food Chemistry, 70:8662-8671. 35797440 10.1021/acs.jafc.2c02490

[207] Watanabe M, Fujihara M, Motoyama T, Kawasaki M, Yamada S, Takamura Y, et al. (2021). Discovery of a "Gatekeeper" Antagonist that Blocks Entry Pathway to Retinoid X Receptors (RXRs) without Allosteric Ligand Inhibition in Permissive RXR Heterodimers. J Med Chem, 64:430-439. 33356247 10.1021/acs.jmedchem.0c01354

[208] Preidis GA, Kim KH, Moore DD (2017). Nutrient-sensing nuclear receptors PPARalpha and FXR control liver energy balance. J Clin Invest, 127:1193-1201. 28287408 10.1172/JCI88893 PMC5373864

[209] Samuel VT, Shulman GI (2018). Nonalcoholic Fatty Liver Disease as a Nexus of Metabolic and Hepatic Diseases. Cell Metab, 27:22-41. 28867301 10.1016/j.cmet.2017.08.002 PMC5762395

[210] Fiorucci S, Rizzo G, Antonelli E, Renga B, Mencarelli A, Riccardi L, et al. (2005). Cross-Talk between Farnesoid-X-Receptor (FXR) and Peroxisome Proliferator-Activated Receptor γ Contributes to the Antifibrotic Activity of FXR Ligands in Rodent Models of Liver Cirrhosis. Journal of Pharmacology and Experimental Therapeutics, 315:58-68. 15980055 10.1124/jpet.105.085597

[211] Zheng W, Lu Y, Tian S, Ma F, Wei Y, Xu S, et al. (2018). Structural insights into the heterodimeric complex of the nuclear receptors FXR and RXR. J Biol Chem, 293:12535-12541. 29934308 10.1074/jbc.RA118.004188 PMC6093246

[212] Jenniskens M, Langouche L, Vanwijngaerden YM, Mesotten D, Van den Berghe G (2016). Cholestatic liver (dys)function during sepsis and other critical illnesses. Intensive Care Med, 42:16-27. 26392257 10.1007/s00134-015-4054-0

[213] Zhang B, Yuan P, Xu G, Chen Z, Li Z, Ye H, et al. (2021). DUSP6 expression is associated with osteoporosis through the regulation of osteoclast differentiation via ERK2/Smad2 signaling. Cell Death Dis, 12:825. 34475393 10.1038/s41419-021-04110-y PMC8413376

[214] Wang XX, Wang D, Luo Y, Myakala K, Dobrinskikh E, Rosenberg AZ, et al. (2018). FXR/TGR5 Dual Agonist Prevents Progression of Nephropathy in Diabetes and Obesity. Journal of the American Society of Nephrology, 29:118-137. 29089371 10.1681/ASN.2017020222 PMC5748904

[215] Younossi ZM, Stepanova M, Nader F, Loomba R, Anstee QM, Ratziu V, et al. (2022). Obeticholic Acid Impact on Quality of Life in Patients With Nonalcoholic Steatohepatitis: REGENERATE 18-Month Interim Analysis. Clin Gastroenterol Hepatol, 20:2050-2058 e2012. 34274514 10.1016/j.cgh.2021.07.020

[216] Siddiqui MS, Van Natta ML, Connelly MA, Vuppalanchi R, Neuschwander-Tetri BA, Tonascia J, et al. (2020). Impact of obeticholic acid on the lipoprotein profile in patients with non-alcoholic steatohepatitis. J Hepatol, 72:25-33. 31634532 10.1016/j.jhep.2019.10.006 PMC6920569

[217] Pockros PJ, Fuchs M, Freilich B, Schiff E, Kohli A, Lawitz EJ, et al. (2019). CONTROL: A randomized phase 2 study of obeticholic acid and atorvastatin on lipoproteins in nonalcoholic steatohepatitis patients. Liver International, 39:2082-2093. 31402538 10.1111/liv.14209

[218] Badman MK, Chen J, Desai S, Vaidya S, Neelakantham S, Zhang J, et al. (2020). Safety, Tolerability, Pharmacokinetics, and Pharmacodynamics of the Novel Non-Bile Acid FXR Agonist Tropifexor (LJN452) in Healthy Volunteers. Clinical Pharmacology in Drug Development, 9:395-410. 31823525 10.1002/cpdd.762 PMC7187203

[219] Camilleri M, Nord SL, Burton D, Oduyebo I, Zhang Y, Chen J, et al. (2020). Randomised clinical trial: significant biochemical and colonic transit effects of the farnesoid X receptor agonist tropifexor in patients with primary bile acid diarrhoea. Aliment Pharmacol Ther, 52:808-820. 32702169 10.1111/apt.15967

[220] Sanyal AJ, Lopez P, Lawitz EJ, Lucas KJ, Loeffler J, Kim W, et al. (2023). Tropifexor for nonalcoholic steatohepatitis: an adaptive, randomized, placebo-controlled phase 2a/b trial. Nat Med, 29:392-400. 36797481 10.1038/s41591-022-02200-8 PMC9941046

[221] Anstee QM, Lucas KJ, Francque S, Abdelmalek MF, Sanyal AJ, Ratziu V, et al. (2023). Tropifexor plus cenicriviroc combination versus monotherapy in non-alcoholic steatohepatitis: Results from the Phase 2b TANDEM study. Hepatology, Publish Ahead of Print. 10.1097/HEP.0000000000000439 PMC10521801 37162151

[222] Ratziu V, Harrison SA, Loustaud-Ratti V, Bureau C, Lawitz E, Abdelmalek M, et al. (2023). Hepatic and renal improvements with FXR agonist vonafexor in individuals with suspected fibrotic NASH. Journal of Hepatology, 78:479-492. 36334688 10.1016/j.jhep.2022.10.023

[223] Loomba R, Noureddin M, Kowdley KV, Kohli A, Sheikh A, Neff G, et al. (2021). Combination Therapies Including Cilofexor and Firsocostat for Bridging Fibrosis and Cirrhosis Attributable to NASH. Hepatology, 73:625-643. 33169409 10.1002/hep.31622

[224] Mo C, Xu X, Zhang P, Peng Y, Zhao X, Chen S, et al. (2023). Discovery of HPG1860, a Structurally Novel Nonbile Acid FXR Agonist Currently in Clinical Development for the Treatment of Nonalcoholic Steatohepatitis. J Med Chem, 66:9363-9375. 37424079 10.1021/acs.jmedchem.3c00456

[225] Fiorucci S, Biagioli M, Baldoni M, Ricci P, Sepe V, Zampella A, et al. (2021). The identification of farnesoid X receptor modulators as treatment options for nonalcoholic fatty liver disease. Expert Opin Drug Discov, 16:1193-1208. 33849361 10.1080/17460441.2021.1916465

[226] Fiorucci S, Biagioli M, Sepe V, Zampella A, Distrutti E (2020). Bile acid modulators for the treatment of nonalcoholic steatohepatitis (NASH). Expert Opin Investig Drugs, 29:623-632. 10.1080/13543784.2020.1763302 32552182

[227] Abdelmalek MF (2021). Nonalcoholic fatty liver disease: another leap forward. Nat Rev Gastroenterol Hepatol, 18:85-86. 33420415 10.1038/s41575-020-00406-0 PMC7791336

[228] Harrison SA, Bashir MR, Lee KJ, Shim-Lopez J, Lee J, Wagner B, et al. (2021). A structurally optimized FXR agonist, MET409, reduced liver fat content over 12 weeks in patients with non-alcoholic steatohepatitis. J Hepatol, 75:25-33. 33581174 10.1016/j.jhep.2021.01.047

[229] Meixiong J, Vasavda C, Snyder SH, Dong X (2019). MRGPRX4 is a G protein-coupled receptor activated by bile acids that may contribute to cholestatic pruritus. Proc Natl Acad Sci U S A, 116:10525-10530. 31068464 10.1073/pnas.1903316116 PMC6535009

[230] Schmidt J, Rotter M, Weiser T, Wittmann S, Weizel L, Kaiser A, et al. (2017). A Dual Modulator of Farnesoid X Receptor and Soluble Epoxide Hydrolase To Counter Nonalcoholic Steatohepatitis. Journal of Medicinal Chemistry, 60:7703-7724. 28845983 10.1021/acs.jmedchem.7b00398

[231] Helmstädter M, Kaiser A, Brunst S, Schmidt J, Ronchetti R, Weizel L, et al. (2021). Second-Generation Dual FXR/sEH Modulators with Optimized Pharmacokinetics. Journal of Medicinal Chemistry, 64:9525-9536. 34165993 10.1021/acs.jmedchem.1c00831

[232] Schierle S, Neumann S, Heitel P, Willems S, Kaiser A, Pollinger J, et al. (2020). Design and Structural Optimization of Dual FXR/PPARδ Activators. Journal of Medicinal Chemistry, 63:8369-8379. 32687365 10.1021/acs.jmedchem.0c00618

[233] Wang XX, Wang D, Luo Y, Myakala K, Dobrinskikh E, Rosenberg AZ, et al. (2018). FXR/TGR5 Dual Agonist Prevents Progression of Nephropathy in Diabetes and Obesity. J Am Soc Nephrol, 29:118-137. 29089371 10.1681/ASN.2017020222 PMC5748904

[234] Schierle S, Brunst S, Helmstädter M, Ebert R, Kramer JS, Steinhilber D, et al. (2021). Development and inβvitro Profiling of Dual FXR/LTA4H Modulators. ChemMedChem, 16:2366-2374. 33856122 10.1002/cmdc.202100118 PMC8453936

[235] Wang S, Lai K, Moy FJ, Bhat A, Hartman HB, Evans MJ (2006). The nuclear hormone receptor farnesoid X receptor (FXR) is activated by androsterone. Endocrinology, 147:4025-4033. 16675527 10.1210/en.2005-1485

[236] Nishimaki-Mogami T, Kawahara Y, Tamehiro N, Yoshida T, Inoue K, Ohno Y, et al. (2006). 5Alpha-bile alcohols function as farnesoid X receptor antagonists. Biochem Biophys Res Commun, 339:386-391. 16300737 10.1016/j.bbrc.2005.11.027

[237] Sayin SI, Wahlstrom A, Felin J, Jantti S, Marschall HU, Bamberg K, et al. (2013). Gut microbiota regulates bile acid metabolism by reducing the levels of tauro-beta-muricholic acid, a naturally occurring FXR antagonist. Cell Metab, 17:225-235. 23395169 10.1016/j.cmet.2013.01.003

[238] Lo B, Holm JP, Vester-Andersen MK, Bendtsen F, Vind I, Burisch J (2020). Incidence, Risk Factors and Evaluation of Osteoporosis in Patients With Inflammatory Bowel Disease: A Danish Population-Based Inception Cohort With 10 Years of Follow-Up. J Crohns Colitis, 14:904-914. 32016388 10.1093/ecco-jcc/jjaa019

[239] Ren L, Song Q, Liu Y, Zhang L, Hao Z, Feng W (2019). Probiotic Lactobacillus rhamnosus GG prevents progesterone metabolite epiallaopregnanolone sulfate-induced hepatic bile acid accumulation and liver injury. Biochem Biophys Res Commun, 520:67-72. 31575408 10.1016/j.bbrc.2019.09.103 PMC6876860

[240] Abu-Hayyeh S, Papacleovoulou G, Lövgren-Sandblom A, Tahir M, Oduwole O, Jamaludin NA, et al. (2013). Intrahepatic cholestasis of pregnancy levels of sulfated progesterone metabolites inhibit farnesoid X receptor resulting in a cholestatic phenotype. Hepatology, 57:716-726. 22961653 10.1002/hep.26055 PMC3592994

[241] Xiong H, Zhang C, Han L, Xu T, Saeed K, Han J, et al. (2022). Suppressed farnesoid X receptor by iron overload in mice and humans potentiates iron-induced hepatotoxicity. Hepatology, 76:387-403. 34870866 10.1002/hep.32270

[242] Anfuso B, Tiribelli C, Adorini L, Rosso N (2020). Obeticholic acid and INT-767 modulate collagen deposition in a NASH in vitro model. Sci Rep, 10:1699. 32015483 10.1038/s41598-020-58562-x PMC6997404

[243] Wen F, Bian D, Wu X, Liu R, Wang C, Gan J (2022). SU5, a new Auraptene analog with improved metabolic stability, ameliorates nonalcoholic fatty liver disease in methionine- and choline-deficient diet-fed db/db mice. Chem Biol Drug Des, 99:504-511. 35040254 10.1111/cbdd.14021

[244] Cao S, Yang X, Zhang Z, Wu J, Chi B, Chen H, et al. (2022). Discovery of a tricyclic farnesoid X receptor agonist HEC96719, a clinical candidate for treatment of non-alcoholic steatohepatitis. Eur J Med Chem, 230:114089. 34998040 10.1016/j.ejmech.2021.114089

[245] Carino A, Marchianò S, Biagioli M, Fiorucci C, Zampella A, Monti MC, et al. (2019). Transcriptome Analysis of Dual FXR and GPBAR1 Agonism in Rodent Model of NASH Reveals Modulation of Lipid Droplets Formation. Nutrients, 11. 31117231 10.3390/nu11051132 PMC6567134

[246] Hernandez ED, Zheng L, Kim Y, Fang B, Liu B, Valdez RA, et al. (2019). Tropifexor-Mediated Abrogation of Steatohepatitis and Fibrosis Is Associated With the Antioxidative Gene Expression Profile in Rodents. Hepatol Commun, 3:1085-1097. 31388629 10.1002/hep4.1368 PMC6672390

[247] Song K, Xu X, Liu P, Chen L, Shen X, Liu J, et al. (2015). Discovery and SAR study of 3-(tert-butyl)-4-hydroxyphenyl benzoate and benzamide derivatives as novel farnesoid X receptor (FXR) antagonists. Bioorg Med Chem, 23:6427-6436. 26337021 10.1016/j.bmc.2015.08.021

[248] Shinozawa E, Amano Y, Yamakawa H, Haba M, Shimada M, Tozawa R (2018). Antidyslipidemic potential of a novel farnesoid X receptor antagonist in a hamster model of dyslipidemia: Comparative studies of other nonstatin agents. Pharmacol Res Perspect, 6:e00390. 29541476 10.1002/prp2.390 PMC5842406

[249] Zhou JX, Li CN, Liu YM, Lin SQ, Wang Y, Xie C, et al. (2022). Discovery of 9,11-Seco-Cholesterol Derivatives as Novel FXR Antagonists. ACS Omega, 7:17401-17405. 35647433 10.1021/acsomega.2c01567 PMC9134407

[250] Teno N, Iguchi Y, Oda K, Yamashita Y, Masuda A, Fujimori K, et al. (2021). Discovery of Orally Active and Nonsteroidal Farnesoid X Receptor (FXR) Antagonist with Propensity for Accumulation and Responsiveness in Ileum. ACS Med Chem Lett, 12:420-425. 33738070 10.1021/acsmedchemlett.0c00640 PMC7957916

[251] Journe F, Laurent G, Chaboteaux C, Nonclercq D, Durbecq V, Larsimont D, et al. (2008). Farnesol, a mevalonate pathway intermediate, stimulates MCF-7 breast cancer cell growth through farnesoid-X-receptor-mediated estrogen receptor activation. Breast Cancer Res Treat, 107:49-61. 17333335 10.1007/s10549-007-9535-6

[252] Song J, Cui ZY, Lian LH, Han X, Hou LS, Wang G, et al. (2020). 20S-Protopanaxatriol Ameliorates Hepatic Fibrosis, Potentially Involving FXR-Mediated Inflammatory Signaling Cascades. J Agric Food Chem, 68:8195-8204. 32662640 10.1021/acs.jafc.0c01978

[253] Lin HR (2012). Triterpenes from Alisma orientalis act as farnesoid X receptor agonists. Bioorg Med Chem Lett, 22:4787-4792. 22683342 10.1016/j.bmcl.2012.05.057

[254] Luan ZL, Ming WH, Sun XW, Zhang C, Zhou Y, Zheng F, et al. (2021). A naturally occurring FXR agonist, alisol B 23-acetate, protects against renal ischemia-reperfusion injury. Am J Physiol Renal Physiol, 321:F617-f628. 34569253 10.1152/ajprenal.00193.2021

[255] Zheng Z, Zhao Z, Li S, Lu X, Jiang M, Lin J, et al. (2017). Altenusin, a Nonsteroidal Microbial Metabolite, Attenuates Nonalcoholic Fatty Liver Disease by Activating the Farnesoid X Receptor. Mol Pharmacol, 92:425-436. 28739572 10.1124/mol.117.108829 PMC5588546

[256] Sun R, Kong B, Yang N, Cao B, Feng D, Yu X, et al. (2021). The Hypoglycemic Effect of Berberine and Berberrubine Involves Modulation of Intestinal Farnesoid X Receptor Signaling Pathway and Inhibition of Hepatic Gluconeogenesis. Drug Metab Dispos, 49:276-286. 33376148 10.1124/dmd.120.000215

[257] Duan X, Meng Q, Wang C, Liu Z, Liu Q, Sun H, et al. (2017). Calycosin attenuates triglyceride accumulation and hepatic fibrosis in murine model of non-alcoholic steatohepatitis via activating farnesoid X receptor. Phytomedicine, 25:83-92. 28190475 10.1016/j.phymed.2016.12.006

[258] Lu Y, Zheng W, Lin S, Guo F, Zhu Y, Wei Y, et al. (2018). Identification of an Oleanane-Type Triterpene Hedragonic Acid as a Novel Farnesoid X Receptor Ligand with Liver Protective Effects and Anti-inflammatory Activity. Mol Pharmacol, 93:63-72. 29162643 10.1124/mol.117.109900

[259] Xu W, Lu C, Zhang F, Shao J, Yao S, Zheng S (2017). Dihydroartemisinin counteracts fibrotic portal hypertension via farnesoid X receptor-dependent inhibition of hepatic stellate cell contraction. Febs j, 284:114-133. 27896916 10.1111/febs.13956

[260] Zhang G, Sun X, Wen Y, Shi A, Zhang J, Wei Y, et al. (2020). Hesperidin alleviates cholestasis via activation of the farnesoid X receptor in vitro and in vivo. Eur J Pharmacol, 885:173498. 32841642 10.1016/j.ejphar.2020.173498

[261] Li Y, Chen H, Ke Z, Huang J, Huang L, Yang B, et al. (2020). Identification of isotschimgine as a novel farnesoid X receptor agonist with potency for the treatment of obesity in mice. Biochem Biophys Res Commun, 521:639-645. 31679693 10.1016/j.bbrc.2019.10.169

[262] Liu M, Zhang G, Song M, Wang J, Shen C, Chen Z, et al. (2020). Activation of Farnesoid X Receptor by Schaftoside Ameliorates Acetaminophen-Induced Hepatotoxicity by Modulating Oxidative Stress and Inflammation. Antioxid Redox Signal, 33:87-116. 32037847 10.1089/ars.2019.7791

[263] Li H, Xi Y, Liu H, Xin X (2022). Gypenosides ameliorate high-fat diet-induced non-alcoholic steatohepatitis via farnesoid X receptor activation. Front Nutr, 9:914079. 36091227 10.3389/fnut.2022.914079 PMC9449333

[264] Shi M, Tang J, Zhang T, Han H (2022). Swertiamarin, an active iridoid glycoside from Swertia pseudochinensis H. Hara, protects against alpha-naphthylisothiocyanate-induced cholestasis by activating the farnesoid X receptor and bile acid excretion pathway. J Ethnopharmacol, 291:115164. 35278607 10.1016/j.jep.2022.115164

[265] Sepe V, Ummarino R, D'Auria MV, Chini MG, Bifulco G, Renga B, et al. (2012). Conicasterol E, a small heterodimer partner sparing farnesoid X receptor modulator endowed with a pregnane X receptor agonistic activity, from the marine sponge Theonella swinhoei. J Med Chem, 55:84-93. 22126372 10.1021/jm201004p

[266] Gu M, Zhang S, Zhao Y, Huang J, Wang Y, Li Y, et al. (2017). Cycloastragenol improves hepatic steatosis by activating farnesoid X receptor signalling. Pharmacol Res, 121:22-32. 28428116 10.1016/j.phrs.2017.04.021

[267] Zhong Y, Chen Y, Pan Z, Tang K, Zhong G, Guo J, et al. (2022). Ginsenoside Rc, as an FXR activator, alleviates acetaminophen-induced hepatotoxicity via relieving inflammation and oxidative stress. Front Pharmacol, 13:1027731. 36278209 10.3389/fphar.2022.1027731 PMC9585238

[268] Farias-Pereira R, Kim E, Park Y (2020). Cafestol increases fat oxidation and energy expenditure in Caenorhabditis elegans via DAF-12-dependent pathway. Food Chem, 307:125537. 31644978 10.1016/j.foodchem.2019.125537

[269] Takahashi M, Kanayama T, Yashiro T, Kondo H, Murase T, Hase T, et al. (2008). Effects of coumestrol on lipid and glucose metabolism as a farnesoid X receptor ligand. Biochem Biophys Res Commun, 372:395-399. 18457666 10.1016/j.bbrc.2008.04.136

[270] Wang F, Zhao C, Tian G, Wei X, Ma Z, Cui J, et al. (2020). Naringin Alleviates Atherosclerosis in ApoE(-/-) Mice by Regulating Cholesterol Metabolism Involved in Gut Microbiota Remodeling. J Agric Food Chem, 68:12651-12660. 33107729 10.1021/acs.jafc.0c05800

[271] Nam SJ, Ko H, Shin M, Ham J, Chin J, Kim Y, et al. (2006). Farnesoid X-activated receptor antagonists from a marine sponge Spongia sp. Bioorg Med Chem Lett, 16:5398-5402. 16905319 10.1016/j.bmcl.2006.07.079

[272] Deng YF, Huang XL, Su M, Yu PX, Zhang Z, Liu QH, et al. (2018). Hypolipidemic effect of SIPI-7623, a derivative of an extract from oriental wormwood, through farnesoid X receptor antagonism. Chin J Nat Med, 16:572-579. 30197122 10.1016/S1875-5364(18)30094-3

[273] Carter BA, Taylor OA, Prendergast DR, Zimmerman TL, Von Furstenberg R, Moore DD, et al. (2007). Stigmasterol, a soy lipid-derived phytosterol, is an antagonist of the bile acid nuclear receptor FXR. Pediatr Res, 62:301-306. 17622954 10.1203/PDR.0b013e3181256492

[274] Sepe V, Bifulco G, Renga B, D'Amore C, Fiorucci S, Zampella A (2011). Discovery of sulfated sterols from marine invertebrates as a new class of marine natural antagonists of farnesoid-X-receptor. J Med Chem, 54:1314-1320. 21309576 10.1021/jm101336m

[275] Cui J, Huang L, Zhao A, Lew JL, Yu J, Sahoo S, et al. (2003). Guggulsterone is a farnesoid X receptor antagonist in coactivator association assays but acts to enhance transcription of bile salt export pump. J Biol Chem, 278:10214-10220. 12525500 10.1074/jbc.M209323200

[276] Liu W, Wong C (2010). Oleanolic acid is a selective farnesoid X receptor modulator. Phytother Res, 24:369-373. 19653193 10.1002/ptr.2948

